# The GATA8-GRF5-XTH9 feed-forward loop regulates cell size in poplar

**DOI:** 10.1093/hr/uhag019

**Published:** 2026-01-20

**Authors:** Yufei Xia, Wenqi Wu, Aoyu Ling, Shenxiu Jiang, Jianghai Shu, Shun Wang, Xinli Xia, Xiangyang Kang

**Affiliations:** State Key Laboratory of Tree Genetics and Breeding, College of Biological Sciences and Technology, Beijing Forestry University, Beijing 100083, China; National Engineering Research Center of Tree Breeding and Ecological Restoration, Beijing Forestry University, Beijing 100083, China; Institute of Crop Sciences, Chinese Academy of Agricultural Sciences, Beijing 100081, China; State Key Laboratory of Tree Genetics and Breeding, College of Biological Sciences and Technology, Beijing Forestry University, Beijing 100083, China; National Engineering Research Center of Tree Breeding and Ecological Restoration, Beijing Forestry University, Beijing 100083, China; State Key Laboratory of Tree Genetics and Breeding, College of Biological Sciences and Technology, Beijing Forestry University, Beijing 100083, China; National Engineering Research Center of Tree Breeding and Ecological Restoration, Beijing Forestry University, Beijing 100083, China; State Key Laboratory of Tree Genetics and Breeding, College of Biological Sciences and Technology, Beijing Forestry University, Beijing 100083, China; National Engineering Research Center of Tree Breeding and Ecological Restoration, Beijing Forestry University, Beijing 100083, China; State Key Laboratory of Tree Genetics and Breeding, College of Biological Sciences and Technology, Beijing Forestry University, Beijing 100083, China; National Engineering Research Center of Tree Breeding and Ecological Restoration, Beijing Forestry University, Beijing 100083, China; State Key Laboratory of Tree Genetics and Breeding, College of Biological Sciences and Technology, Beijing Forestry University, Beijing 100083, China; State Key Laboratory of Tree Genetics and Breeding, College of Biological Sciences and Technology, Beijing Forestry University, Beijing 100083, China; National Engineering Research Center of Tree Breeding and Ecological Restoration, Beijing Forestry University, Beijing 100083, China

## Abstract

Although triploid poplars have larger cells and leaves than their diploid counterparts, the molecular mechanisms underlying this disparity remain elusive. Here, we found that *PpnGATA8* and *PpnGRF5* were significantly upregulated in triploid poplars through differential gene expression analysis between diploid and triploid poplars. Furthermore, through genetic transformation in poplar, it was found that both *PpnGATA8* and *PpnGRF5* positively regulated poplar cell size, resulting in increased leaf size and improved photosynthetic efficiency. RNA-sequencing of *PpnGATA8-*overexpressing poplars showed that PpnGATA8 promotes expression of *PagGRF5* and *PagXTH9*. Yeast one-hybrid system, electrophoretic mobility shift assay, and dual-luciferase assay were employed to substantiate that PpnGATA8 directly regulated *PagGRF5* and *PagXTH9* expression. Meanwhile, PpnGRF5 positively regulates the expression of *PagXTH9*. Poplar protoplast cotransformation assays further proved that coexpression of *PpnGATA8* and *PpnGRF5* had the strongest effect on promoting *PagXTH9* expression. Moreover, overexpression of *PpnXTH9* also significantly increased poplar cell and leaf size. Therefore, *GATA8*, *GRF5*, and *XTH9* formed a feed-forward regulatory loop to regulate plant cell size. Our results are of major significance for revealing the molecular regulatory mechanisms of plant cell size and leaf development, especially the genetic basis of giant variation in cells and leaves in polyploid plants.

## Introduction

Genome polyploidization is common during the evolution of many plant species [1]. Many plants are polyploid, including many varieties of important crops such as hexaploid wheat, tetraploid cotton, and potatoes [2–4]. Although crops such as rice and soybeans are diploid, they have also experienced at least one whole-genome doubling event during their evolution [5–7]. Chromosome doubling can create new varieties that grow rapidly; have large leaves, flowers, and fruits; high nutritional content, and strong adaptability to the environment, such as triploid poplar, triploid and tetraploid *Arabidopsis thaliana* and rice [8–10]. Of course, not all polyploid plants show gigantism. Compared with diploid plants, tetraploid plants of *Populus*, *Citrus limonia*, and *Ziziphus jujuba* Mill. var. *spinosa* have relatively short internodes resulting in relatively short plants [11–13]. However, these polyploid plants generally have cells that grow larger as ploidy increases [9, 11, 14]. So far, the mechanism of the formation of polyploid cell gigantism in plants has not been revealed.

The control of plant cell size is achieved through a coupling of cell division and single-cell growth. Once cell proliferation stops, cells begin to expand, which becomes the main driver of organ growth [15]. Plant cell size depends on the expression of cell growth, hormones, cell cycle-related genes, and cell wall and cytoplasm biosynthesis-related genes [16–19]. *XTHs* are key enzymes in cell wall remodeling, which can participate in cell elongation and expansion by controlling the elasticity and extensibility of cells [20]. Among them, *XTH9*, as a xyloglucan endotransglycosylase (*XTHs*), can affect cell expansion, secondary wall formation, and lateral root development [21–23], but its upstream regulatory factors are currently only reported to be *CUC2*, *OBP4*, and others [23, 24]. So far, no research has been reported on the regulation of *XTH9* by *GRF5* and *GATA8* transcription factors.

Feed-forward loop (FFL) is a common gene network regulation model [25]. FFL consists of two input regulatory factors A and B and one output factor C. A regulates B, and A and B jointly regulate the target factor C. This regulatory mode ensures that the system can respond flexibly and accurately when faced with changes in external signals [26, 27], significantly accelerating/slowing down the accumulation/reduction of downstream genes [28, 29]. The above patterns have been reported in species such as *Arabidopsis* and soybean [30, 31]. As a basic module of plant gene regulatory network, FFL plays an important role in fine-tuning gene expression and physiological responses. *GATA* and *GRF* families are important transcription factor families that regulate cell size. *GATA* family members are involved in regulating photosynthesis, stomatal formation, and the development of roots, leaves, and flowers [32]. *Arabidopsis* or poplar seedlings overexpressing *GNC*, *GNL*, *GATA15*, and *GATA17* have longer hypocotyls and more chlorophyll accumulation than wild-type (WT) plants, which in turn affects photosynthesis [33, 34]. Studies have indicated that *GATA* transcription factors can directly affect plant cell size and photosynthesis. For example, overexpression (OE) of *GATA25* and *GNC* can lead to cell enlargement [35–37]. *GATA8* regulates cell differentiation and biomass accumulation in *Arabidopsis* and rice [38, 39]. Most genes in the *GRF* gene family can affect cell growth and plant vegetative growth, such as *GRF1*, *GRF2*, *GRF3*, *GRF5*, and *GRF15*, which can increase cell size or leaf area [40–43], thereby affecting plant photosynthesis, etc [44, 45]. So far, there has been no research report on *GATA8* directly regulating *GRF5* and forming FFL regulatory patterns with functional genes that determine cell size.

Previous research found that compared with diploid poplars, differential genes positively related to cell growth were significantly upregulated in triploid poplars [42]. Therefore, can we explore the formation mechanism during the development of polyploid cells through differential gene expression? The research results are positive. This study used diploid and triploid poplar as materials and found that *PpnGATA8* and *PpnGRF5* were significantly highly expressed in apical buds of triploid poplar. Based on this, *PpnGATA8* and *PpnGRF5* were cloned from *Populus* section *Tacamahaca*, and the phenotypic determination and verification of molecular regulation relationships of transgenic plants proved that OE of *PpnGATA8* and *PpnGRF5* can enlarge leaf area and cell size of diploid poplars, which is consistent with the phenotype of triploid poplars. It was found that *GATA8* and *GRF5* can simultaneously regulate *XTH9* and form FFL regulation pattern. This study provides critical theoretical insights into the molecular mechanisms underlying giant variation in plant polyploid cells.

## Results

### Variation of leaf cell size and photosynthetic parameters in triploid poplars

**Figure 1:**
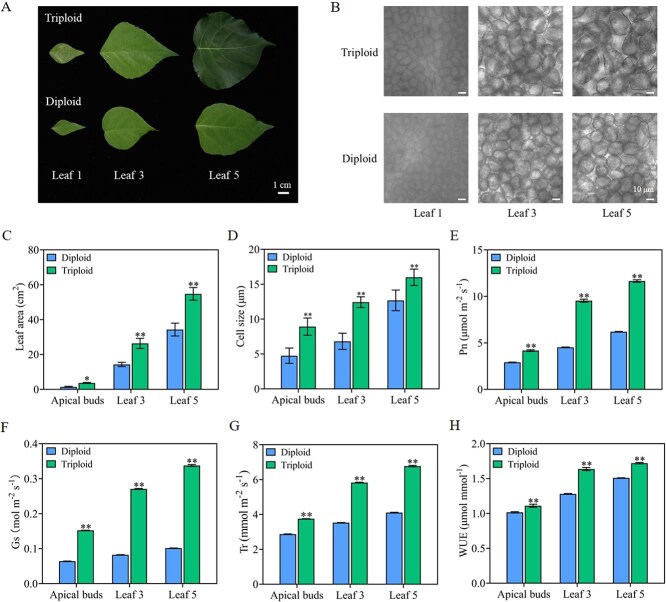
Analysis of phenotypes in diploid and triploid poplars. (A) leaf and (B) cell size phenotypes in diploid and triploid poplars. (C) Leaf area, (D) cell size, (E) Pn, (F) Gs, (G) Tr, and (H) WUE of diploid and triploid poplars. Values represent the mean ± SD (*n* ≥ 3). ^*^P < 0.05; ^**^P < 0.01.

Previous studies have demonstrated that triploid poplars possess significantly larger cell sizes than diploid poplars [42]. Measurements of the leaf phenotypes and photosynthetic parameters of diploid and triploid poplars revealed that triploid poplars exhibited significantly larger leaf area and cell size than diploid poplars across different tissues (Fig. 1A–D). The net photosynthetic rate (Pn), stomatal conductance (Gs), transpiration rate (Tr), and water use efficiency (WUE) of triploid poplar were higher than those of diploid poplar in apical buds and leaves at the third and fifth leaf positions (Fig. 1E–H). These results indicate that the larger cell and leaf size of triploid poplar may be the key reason for its higher rate of photosynthesis.

### Identification of candidate genes that control cell size in triploid poplar

To elucidate the molecular basis underlying the differences in cell and leaf size between diploid and triploid poplar, we first conducted an expression trend analysis using transcriptome data from the apical buds (Dip. 1), 17th leaves (Dip. 9), and 25th leaves (Dip. 13) of both diploid and triploid poplars. The results revealed that the majority of genes exhibited significantly higher expression levels in the apical buds compared with the other tissues (Fig. S1A and B). Furthermore, genes that were highly expressed in the apical buds were intersected with growth- and development-related differentially expressed genes (DEGs) identified in diploid and triploid poplars, and a correlation network was subsequently constructed. The analysis revealed that *GATA8* exhibited the highest network weight and occupied a central position within the network (Fig. S1C). It was speculated that *GATA8* might be related to the regulation of cell and leaf size in triploid poplars.

**Figure 2:**
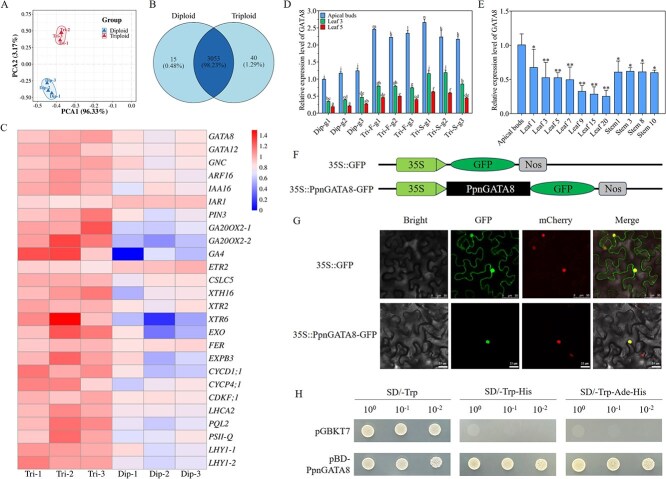
Transcriptome analysis of triploid and diploid poplar, GATA8 expression pattern analysis, and detection of subcellular localization and transcriptional activation activity of *PpnGATA8*. (A) Principal component analysis (PCA) of triploid and diploid poplar transcriptome sequencing samples. (B) Venn diagram of DEGs between diploid and triploid poplar. (C) Heatmap of the expression levels of growth-related genes in triploid and diploid poplars. The data were normalized. Dip, Diploid; Tri, Triploid. (D) Expression level of *PpnGATA8* in three different genotypes of diploid (Dip) and triploid (Tri) poplars. F, first division restitution gametes; S, second division restitution gametes; g1, genotype 1. (E) Expression patterns of *PpnGATA8* in different tissues of poplars. Values represent the mean + SD (*n* ≥ 3). ^*^P < 0.05; ^**^P < 0.01. (F) structure diagram of 35S::PpnGATA8-GFP and 35S::GFP vectors. (G) Subcellular localization of the PpnGATA8 protein. mCherry is a red fluorescent protein localized in the nucleus. (H) Transactivation assay of PpnGATA8.

Since cell size formation mainly occurs in young tissues, we subsequently selected transcriptome data from diploid and triploid poplar apical buds for further analysis. Principal component analysis revealed that the biological replicates of each sample displayed a high degree of similarity, suggesting that the transcriptome data were reliable and appropriate for further analysis (Fig. 2A). The triploid and diploid poplars shared 3053 DEGs (Fig. 2B; Table S1). In apical buds of triploid poplar, the expression levels of 1295 genes were significantly upregulated and 1813 genes were significantly downregulated when compared with diploid poplar (Fig. S2). Here, DEGs in triploid and diploid poplar were analyzed, and many genes related to cell growth and development were found to be significantly differentially expressed. For example, *GATA8* and *GNC* of the *GATA* family were all highly expressed in triploids; *XTH16*, *XTR2*, *XTR6*, *EXO*, and *EXPB3*, which are related to cell wall biosynthesis, were also differentially expressed (Fig. 2C; Table S2). A correlation network analysis was performed on the above-mentioned growth and development-related genes, and it was found that *GATA8* ranked high in the correlation network and was located in the core position (Fig. S1D). Furthermore, through a comparison of amino acid sequences, it was found that PpnGATA8 had a similar structural domain to PtrGATA8 in *Populus trichocarpa* and AtGATA8 in *A. thaliana*, with a high level of sequence similarity (Fig. S3). It was speculated that *PpnGATA8* might have similar functions. In diploid and triploid poplar, whether *GATA8* plays a key role in regulating cell size will be further studied.

### *PpnGATA8* expression pattern analysis and detection of transcriptional activation activity

Based on transcriptome data, *PpnGATA8* was highly expressed in triploid poplars (Fig. 2C). Subsequently, three different tissues were collected from diploid and triploid poplars to verify the expression pattern of the *PpnGATA8*. It was found that *PpnGATA8* had roughly similar expression patterns in diploid and triploid poplar. The expression of *PpnGATA8* was higher in apical buds than in other tissues (Fig. 2D). The expression level of *PpnGATA8* decreased with an increase in leaf number (Fig. 2D), suggesting *PpnGATA8* might mainly function in young tissues. Compared with diploid poplar, *PpnGATA8* was significantly highly expressed in three different tissues of different genotypes of triploid poplars (Fig. 2D), indicating that the expression of *PpnGATA8* in triploid poplar leaves had a dose effect.

To study the expression pattern of *PpnGATA8* in different tissues, apical buds, leaves at the first, third, fifth, seventh, ninth, 15th, and 20th leaf position, and the first, third, eighth, and 10th internodes were collected from 3-month-old 84 K (*Populus alba* × *Populus glandulosa*) poplars for reverse transcription-quantitative polymerase chain reaction (RT-qPCR) detection. *PpnGATA8* had the highest level of expression in apical buds. As the leaf position increased, its level of expression gradually decreased, but its level of expression in stem segments was generally high (Fig. 2E).

Next, *PpnGATA8* was cloned from triploid *Populus* section *Tacamahaca*. To determine the protein localization of *PpnGATA8*, 35S::*PpnGATA8*-GFP and 35S::GFP constructs were generated (Fig. 2F). The 35S::*PpnGATA8*-GFP protein clearly colocalized with mCherry red fluorescent protein, indicating that the *PpnGATA8* protein was localized in the nucleus, while the 35S::GFP control protein was observed in both the nucleus and cytoplasm (Fig. 2G). In activity detection assays, transformants harboring GALBD-*PpnGATA8* exhibited robust growth on both SD/–Trp/–His and SD/–Trp/–Ade/–His media, while the negative control pGBKT7 vector failed to grow (Fig. 2H), demonstrating that PpnGATA8 has transcriptional activation activity in yeast cells.

### PpnGATA8 regulates leaf development and vegetative growth

To examine whether the enlargement of cells and leaves in triploid poplars results from increased *PpnGATA8* expression, we overexpressed *PpnGATA8* in *A. thaliana*. The results indicated that *A. thaliana* overexpressing *PpnGATA8* had thicker and larger leaves compared to WT (Fig. S4A and B).

**Figure 3:**
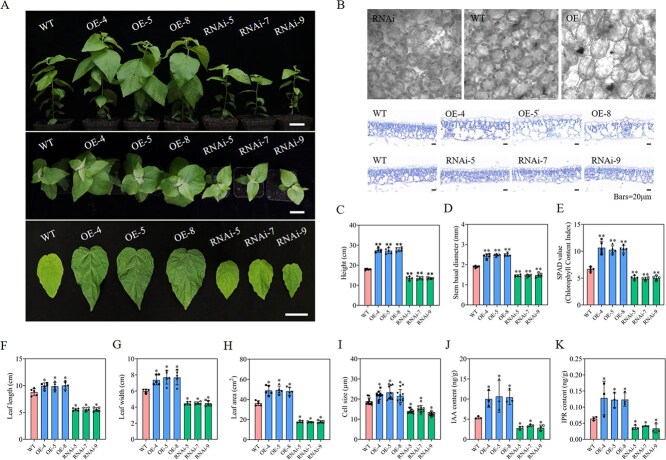
*PpnGATA8* affects plant cell and leaf size as well as vegetative growth in poplar. (A) phenotypes of 1-month-old poplars and the fifth leaf position of OE and RNAi plants expressing *PpnGATA8*. Bars = 5 cm; (B) mesophyll and epidermal cells of OE and RNAi lines. (C) plant height, (D) basal diameter, (E) SPAD value, (F) leaf length of fifth leaf position, (G) leaf width of fifth leaf position, (H) leaf area of fifth leaf position, (I) cell size, (J) IAA content, and (K) IPR content of OE and RNAi poplars. Values represent the mean ± SD (*n* ≥ 3). ^*^P < 0.05, ^**^P < 0.01; determined by one-way ANOVA.

Meanwhile, OE and RNA interference expression (RNAi) transgenic lines of *PpnGATA8* were constructed in 84 K poplars. Ten independent transgenic lines overexpressing *PpnGATA8* and 10 independent transgenic lines interfering with the expression of *PpnGATA8* were obtained (Fig. S5A and B). OE-4, OE-5, and OE-8 along with RNAi-5, RNAi-7, and RNAi-9 with intermediate expression levels were chosen for further investigation (Fig. 3A).

First, the growth conditions of transgenic and WT plants were observed, and it was shown that the plant height and basal diameter of OE plants were significantly larger than those of WT plants (Fig. 3C and D), and their relative chlorophyll content (SPAD value) as well as their leaf length, width, and area were also significantly greater than those of WT plants (Fig. 3E–H), while the RNAi poplars showed the opposite phenotype to OE plants (Fig. 3C–H). The fresh and dry weights of the leaves of OE plants were also significantly greater than those of WT plants (Fig. S6). Subsequently, it was found that the size of the mesophyll cells in the leaves of the OE poplars was significantly larger than that of the WT poplars, while that of the RNAi poplars was significantly smaller than that of the WT poplars (Fig. 3B and I). The number of cells per unit area of the OE lines was significantly less than that of the WT plants, but the total number of cells was significantly greater than that of the WT, while the RNAi plants showed the opposite phenotype to that of OE (Fig. S7A and B). Furthermore, paraffin sections of the fifth internode stem segments from WT, OE, and RNAi plants were analyzed. The results showed that stem cell size was significantly increased in PpnGATA8-OE plants compared with WT, whereas stem cell size was significantly reduced in PpnGATA8-RNAi plants (Fig. S8). Considering that both auxin and cytokinin influence cell size, we measured their contents. The results indicated that indole-3-acetic acid (IAA) and isopentenyladenosine (IPR) contents were markedly elevated in OE poplars compared with WT poplars, whereas they were markedly reduced in RNAi poplars relative to WT poplars (Fig. 3J and K).

### PpnGATA8 affects cell size and growth by influencing the expression of *PagGRF5* and *PagXTH9* in poplar

**Figure 4:**
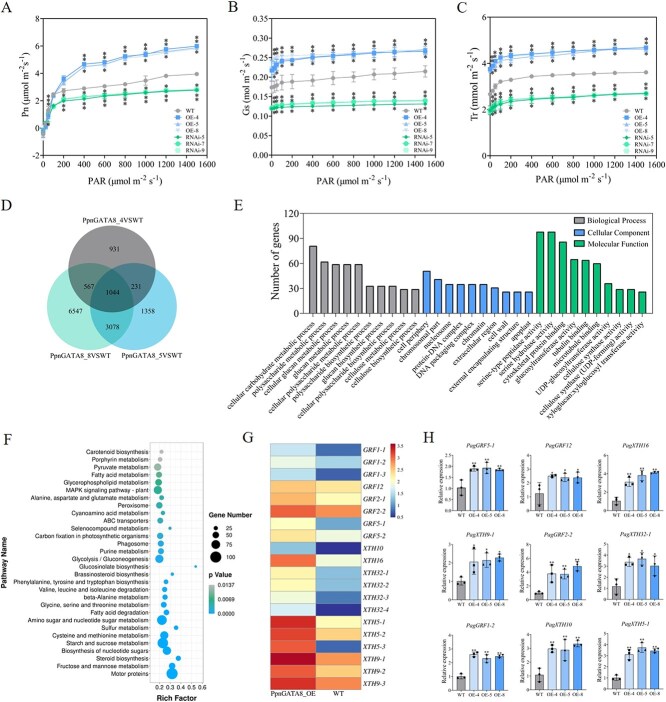
*PpnGATA8* affects plant cell size and photosynthesis by affecting the expression of GRFs and XTHs. (A) *Pn*—light curve. (B) *Gs*—light curve. (C) *Tr*—light curve. (D) Venn diagram of three OEs. (E) GO enrichment analysis. (F) KEGG enrichment analysis. (G) Metric fragments per kilobase of feature per million mapped reads (FPKM) values of *GRF* and XTH family genes in OE and WT poplars. The gene expression levels shown in the figure represent the log10-transformed values of the original data. (H) The expression levels of *GRFs* and *XTHs* were detected by RT-qPCR in OE and WT poplars. Values represent the mean ± SD (*n* = 3). ^*^P < 0.05, ^**^P < 0.01.

Considering the above phenotypic changes, changes in cell size should also have an impact on plant photosynthesis; therefore, the gas exchange parameters of leaves of different lines were measured. It was found that under different photosynthetically active radiation, the *Pn*, *Gs*, and *Tr* of the OE poplars were significantly higher than those of the WT poplars, while those of the RNAi poplars were significantly lower than those of the WT poplars (Fig. 4A–C).

Subsequently, the fifth leaves of OE-4, OE-5, OE-8, and WT plants were collected for transcriptome sequencing. A total of 5769 upregulated and 4622 downregulated expression genes were found between OE and WT poplars (Fig. S9; Table S3). In addition, there were 1044 shared DEGs between the three OE and WT lines (Fig. 4D). We randomly selected 10 genes that were significantly upregulated in OE and performed RT-qPCR validation in OE and RNAi poplars. The results showed that all 10 genes were significantly upregulated in OE poplars and downregulated in RNAi poplars (Fig. S10). Gene ontology (GO) enrichment analysis showed that DEGs were significantly enriched in cellular carbohydrate and cellular glucan metabolic processes as well as in cell wall development, cytoskeletal protein binding, and glucosyltransferase activity (Fig. 4E; Table S4). Kyoto Encyclopedia of Genes and Genomes (KEGG) enrichment analysis showed that DEGs were significantly enriched in pathways such as carbon fixation in photosynthetic organisms and the biosynthesis of phenylalanine, tyrosine, and tryptophan (Fig. 4F; Table S5). Some genes of the GRF and XTH families were significantly highly expressed in OE poplars, such as *PagGRF1*, *PagGRF12*, *PagGRF2*, *PagGRF5*, *PagXTH10*, *PagXTH16*, *PagXTH32*, *PagXTH5*, and *PagXTH9* (Fig. 4G; Table S6). Meanwhile, genes related to the cell cycle, cell growth, or cell wall expansion were generally expressed at higher levels in OE poplars (Fig. S11). RT-qPCR results showed that *PagGRF5-1*, *PagXTH9-1*, *PagGRF1-2*, *PagGRF12*, *PagGRF2-2*, *PagXTH10*, *PagXTH16*, *PagXTH32-1*, and *PagXTH5-1* were all significantly highly expressed in OE leaves (Fig. 4H). Previous studies have reported that *GRF5* could significantly increase cell size in poplar [42], and *XTH9* could also promote cell expansion [21, 22]. Therefore, it was speculated that *PpnGATA8* might increase cell size and the rate of photosynthesis by promoting the expression of *PagGRF5* and *PagXTH9*.

### PpnGATA8 directly binds to the promoters of *PagGRF5* and *PagXTH9* and promotes their expression

**Figure 5:**
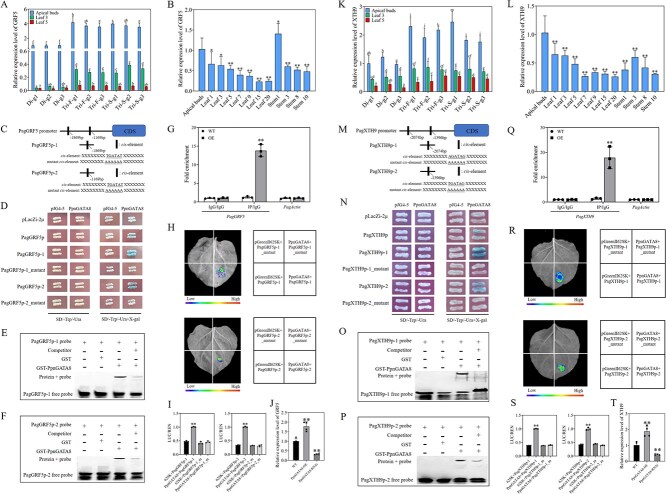
PpnGATA8 directly binds to the *PagGRF5* and *PagXTH9* promoters and activates their expression. (A) Expression level of *PpnGRF5* in three different genotypes of diploid (Di) and triploid (Tri) poplars. (B) Expression patterns of *PpnGRF5* in different tissues of poplars. (C) Putative *PpnGATA8-*binding element in the *PagGRF5* promoter; the absolute value of each number represents the distance to the start codon. (D) Y1H experiments showing that *PpnGATA8* directly binds to motifs in the *PagGRF5* promoter; blue colonies indicate that *PpnGATA8* is strongly associated with a specific promoter fragment. (E, F) EMSAs showing that the GST-PpnGATA8 recombinant protein binds to biotin-labeled probes of *PagGRF5*-1 and -2. (G) ChIP-qPCR analysis. The abundance of the *PagGRF5* promoter sequence was obtained for chromatin preparation before immunoprecipitation (Input), and was immunoprecipitated with GFP polyclonal antibody (ChIP) and IgG. (H) Transient expression analysis showing that *PpnGATA8* directly promotes the expression of *PagGRF5*-1 and -2. (I) Dual-luciferase assay was used to determine the expression of *PagGRF5*-1::LUC and *PagGRF5*-2::LUC; the LUC/REN ratio represents the relative activity of *PagGRF5*-1 and -2 promoters. (J) Expression of *GRF5* in OE and RNAi poplar leaves. (K) Expression level of *XTH9* in diploid and triploid poplars. (L) Expression patterns of *XTH9* in different tissues of poplars. (M) Putative *PpnGATA8*-binding element in the *PagXTH9* promoter. (N) Y1H experiments showing that *PpnGATA8* directly binds to motifs in the *PagXTH9* promoter; blue colonies indicate that *PpnGATA8* is strongly associated with a specific promoter fragment. (O, P) EMSAs showing that the GST-PpnGATA8 recombinant protein binds to biotin-labeled probes of *PagXTH9*-1 and -2. (Q) The abundance of the *PagXTH9* promoter sequence was obtained for chromatin preparation before immunoprecipitation (Input), and was immunoprecipitated with GFP polyclonal antibody (ChIP) and IgG. (R) Transient expression analysis showing that *PpnGATA8* directly promotes the expression of *PagXTH9*-1 and -2. During the image visualization process, we normalized the luminescence value of the empty carrier control group to zero. (S) Dual-luciferase assay was used to determine the expression of *PagXTH9*-1::LUC and *PagXTH9*-2::LUC. (T) Expression levels of *XTH9* in OE and RNAi poplar leaves. Values shown are mean ± SD (*n* ≥ 3). ^**^P < 0.01.

Since *PagGRF5* and *PagXTH9* were significantly upregulated in plants with OE of *PpnGATA8* (Fig. 4G and H), it was hypothesized that *PpnGATA8* directly promoted *PagGRF5* and *PagXTH9* expression. First, it was found that the expression trend of *GRF5* in diploid and triploid poplars was very similar to that of *GATA8*, with the highest expression in apical buds and significantly higher expression in triploids (Fig. 5A). In addition, *GRF5* was expressed at the highest level in apical buds (Fig. 5B).

To test whether a direct regulatory relationship exists between *PpnGATA8* and *PagGRF5*, the promoter region of the *PagGRF5* was analyzed. The results showed this promoter region contained GATA-specific binding elements (Table S7), and the *cis*-element (TGATAT) of the *PpnGATA8* binding site was found to exist at 1869 and 1169 bp upstream of the *PagGRF5* promoter. A 2-kb promoter region and an ~150-bp flanking region of this motif (300 bp in total) were amplified and named *PagGRF5*p, *PagGRF5*p-1 (−1869 bp site), and *PagGRF5*p-2 (−1169 bp site) (Fig. 5C). In addition, *PpnGATA8* was found to stably interact with the TGATAT motif in the *PagGRF5* promoter. Mutation of this *cis*-element in the *PagGRF5* promoter fragment eliminated *PpnGATA8* binding, indicating that this interaction was genuine (Fig. 5D). Electrophoretic mobility shift assays (EMSAs) demonstrated that PpnGATA8 specifically bound to the promoter containing the TGATAT sequence, and the binding intensity became weaker after adding competitive probes (Fig. 5E and F). Chromatin Immunoprecipitation–quantitative PCR (ChIP-qPCR) analysis further revealed a significant enrichment of *PagGRF5* in *PpnGATA8*-overexpressing poplars, showing a >10-fold increase compared with WT poplars (Fig. 5G). Next, we examined whether *PpnGATA8* could directly regulate the transcription of *PagGRF5* using a dual-luciferase reporter assay. It was found that in the presence of *PpnGATA8* and *PagGRF5*p-1, *PpnGATA8* and *PagGRF5*p-2, the *PagGRF5*p-1::LUC or *PagGRF5*p-2::LUC reporter gene was activated, but not activated otherwise (Fig. 5H). In addition, by detecting the LUC/REN value, the same conclusion was found (Fig. 5I). We selected materials with successful OE and silent expression of *GATA8* to detect the expression level of *GRF5* (Fig. S12). Subsequently, the expression of *GRF5* was detected in *PpnGATA8*-OE and *PpnGATA8*-RNAi poplar leaves; it was found that its expression was significantly higher in *PpnGATA8*-OE plants, while its expression was significantly reduced in *PpnGATA8*-RNAi plants (Fig. 5J). Therefore, the above results proved that *PpnGATA8* could directly bind to the promoter of *PagGRF5* and promote the expression of *PagGRF5*.

The expression of *XTH9* was still detected in diploid and triploid poplars, and it was found that it exhibited an expression pattern similar to that of *GATA8* (Fig. 5K), and *XTH9* was also expressed at the highest level in apical buds (Fig. 5L). Based on the expression levels in diploid and triploid poplars, and the significant upregulation of *XTH9* in *GATA8*-overexpressing poplars detected in Fig. 4H, it was speculated that *GATA8* might directly regulate *XTH9* expression. Subsequently, the 3-kb proximal promoter region upstream of the *PagXTH9* translation start site was analyzed and found to have GATA and GRF-specific binding elements (Table S8). The *cis*-elements (AGATAG and TGATAG) of the *PpnGATA8-*binding site were located 2074 and 1396 bp upstream of the *PagXTH9* promoter, respectively. The 3-kb promoter of *PagXTH9* (*PagXTH9*p) and the short fragments containing the *cis*-acting elements (*PagXTH9*p-1 and *PagXTH9*p-2) were amplified (Fig. 5M). Then, yeast one-hybrid (Y1H) assays showed that *PpnGATA8* could bind to the AGATAG and TGATAG motifs in the *PagXTH9* promoter region (Fig. 5N). Further EMSA experiments demonstrated that PpnGATA8 could specifically bind to promoter fragments in *PagXTH9* containing AGATAG and TGATAG sequences (Fig. 5O and P). ChIP–qPCR assays further demonstrated that *PagXTH9* was significantly enriched in *PpnGATA8*-overexpressing plants (Fig. 5Q). It was found that the *PagXTH9*p-1::LUC or *PagXTH9*p-2::LUC reporter genes were significantly activated only when *PpnGATA8* and *PagXTH9*p-1 or *PpnGATA8* and *PagXTH9*p-2 were present simultaneously (Fig. 5R). The same conclusion was made after detecting the LUC/REN value (Fig. 5S). We selected materials with successful OE and silent expression of *PpnGATA8* to detect the expression level of *PagXTH9* (Fig. S12). It was found that its expression level was significantly increased in OE poplar leaves, while its expression level was significantly decreased in RNAi poplar leaves (Fig. 5T). The above results illustrated that *PpnGATA8* could directly bind to the promoter of *PagXTH9* and promote the expression of *PagXTH9*.

### PpnGRF5 regulates poplar cell size by directly promoting the expression of *PagXTH9*

**Figure 6:**
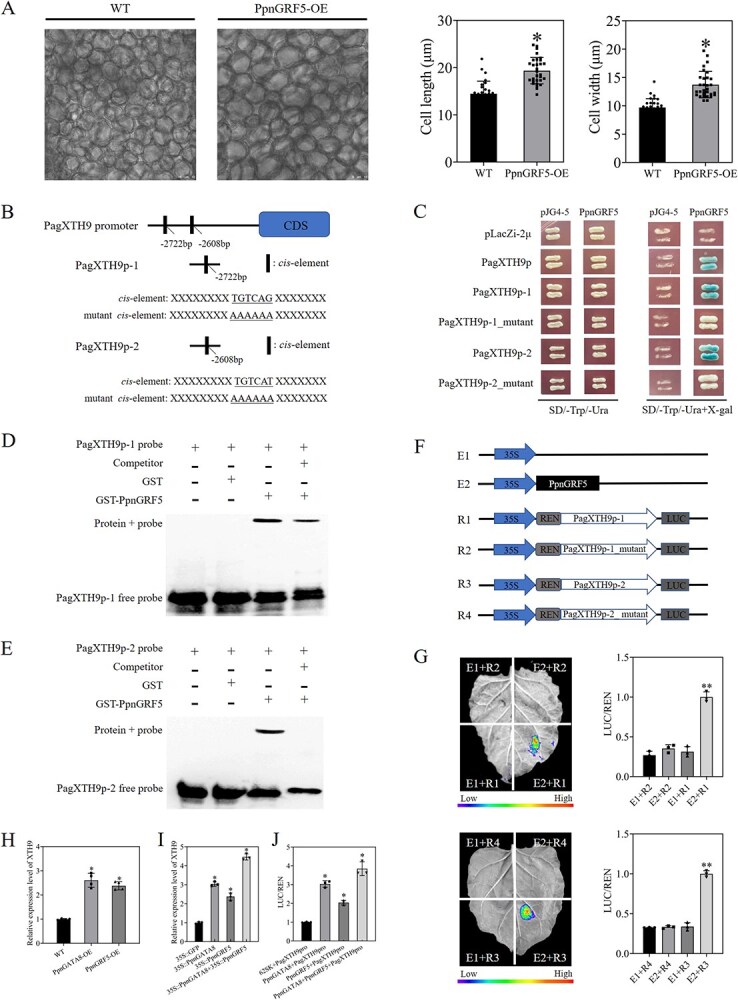
PpnGRF5 binds directly to the *PagXTH9* promoter and activates its expression, thereby increasing cell size. (A) Cell size phenotypes of *PpnGRF5*-overexpressing and WT poplar. (B) Putative *PpnGRF5* binding element in the *PagXTH9* promoter. (C) Y1H experiments showing that *PpnGRF5* directly binds to motifs in the *PagXTH9* promoter. (D, E) EMSAs showing that PpnGRF5 protein binds to biotin-labeled probes of *PagXTH9*-1 and -2; the binding sites are speculated to be TGTCAG and TGTCAT. (F) Schematic representation of effector and reporter vector structures. (G) Transient expression analysis showing that *PpnGRF5* directly promotes *PagXTH9*-1 and *PagXTH9*-2 expression. Dual luciferase assays used to determine the expression of *PagXTH9*-1::LUC and *PagXTH9*-2::LUC. (H) The expression level of *PagXTH9* in *PpnGATA8-* or *PpnGRF5*-overexpressing plants was detected. (I) The expression level of *PagXTH9* in poplar protoplasts transformed with empty vector or 35S-*PpnGATA8*-GFP or 35S-*PpnGRF5*-GFP alone and cotransformed with 35S-*PpnGATA8*-GFP and 35S-*PpnGRF5*-GFP was detected. (J) *PagXTH9* promoter activity upon coexpression of *PpnGATA8* and *PpnGRF5* or individual expression of each factor in tobacco leaves. Values shown are mean ± SD (*n* ≥ 3). ^**^P < 0.01; ^*^P < 0.05.

Examination of the phenotype of *PpnGRF5-*overexpressing plants revealed that the cell size of these plants was significantly larger than those of WT plants (Fig. 6A). Because it had a similar expression pattern to *XTH9*, it was hypothesized that overexpressing *PpnGRF5* increased cell size by regulating the expression of *XTH9*. The *cis*-elements (TGTCAG and TGTCAT) of the *PpnGRF5*-binding site were found to exist at 2722 and 2608 bp upstream of the *PagXTH9* promoter. About 150-bp flanking regions of this motif were amplified and named *PagXTH9*p-1 (2722 bp site) and *PagXTH9*p-2 (2608 bp site) (Fig. 6B). Y1H experiments revealed that *PpnGRF5* stably bound to the TGTCAG and TGTCAT motifs within the proximal promoter of *PagXTH9* (Fig. 6C). Furthermore, EMSAs demonstrated that PpnGRF5 specifically bound to promoter fragments containing TGTCAG and TGTCAT sequences (Fig. 6D and E). Next, the effector and reporter plasmids were constructed (Fig. 6F). It was found that the *PagXTH9*p-1::LUC or *PagXTH9*p-2::LUC reporter gene was activated in the presence of *PpnGRF5* and *PagXTH9*p-1, *PagGRF5* and *PagXTH9*p-2, but not otherwise (Fig. 6G). Therefore, this study proved that *PpnGRF5* could directly bind to the promoter of *PagXTH9* and promote the expression of *PagXTH9*, thereby increasing the size of poplar cells.

To prove the above conclusions, we further verified the above regulatory relationship in poplar. We selected materials that were successfully overexpressed with *PpnGATA8* or *PpnGRF5* to detect the expression level of *PagXTH9* (Figs S12 and S13). It was found that the expression level of *PagXTH9* in *PpnGATA8-* and *PpnGRF5-*overexpressing plants was significantly higher than that in WT (Fig. 6H). Subsequently, we successfully transformed the *PpnGATA8* and *PpnGRF5* OE vectors into poplar leaf protoplasts. It was found that when *PpnGATA8* and *PpnGRF5* were overexpressed simultaneously, the expression of *XTH9* was significantly upregulated (Fig. 6I). Dual-luciferase reporter assays showed that the *PagXTH9* promoter activity was significantly enhanced when *PpnGATA8* and *PpnGRF5* were coexpressed in tobacco leaves compared with individual expression of either gene (Fig. 6J). To clarify whether there was a direct protein–protein interaction between PpnGATA8 and PpnGRF5, yeast two-hybrid and split luciferase complementation assays were conducted on PpnGATA8 and PpnGRF5. The results showed that there was no direct protein–protein interaction between PpnGATA8 and PpnGRF5 (Fig. S14A and B). The results indicated that *PpnGATA8* and *PpnGRF5* activated *PagXTH9* expression by independently binding to different regions of the promoter, rather than by forming a protein complex for synergistic regulation. When *PpnGATA8* and *PpnGRF5* were present simultaneously, the promoting effect of *PpnGATA8* on *PpnGRF5* expression further enhanced the activation effect of *PpnGRF5* on *PagXTH9*, thereby forming an FFL at the transcriptional regulation level, ultimately leading to a significant increase in *PagXTH9* expression levels.

### *PpnXTH9* significantly increases poplar leaf and cell size and promotes plant growth

**Figure 7:**
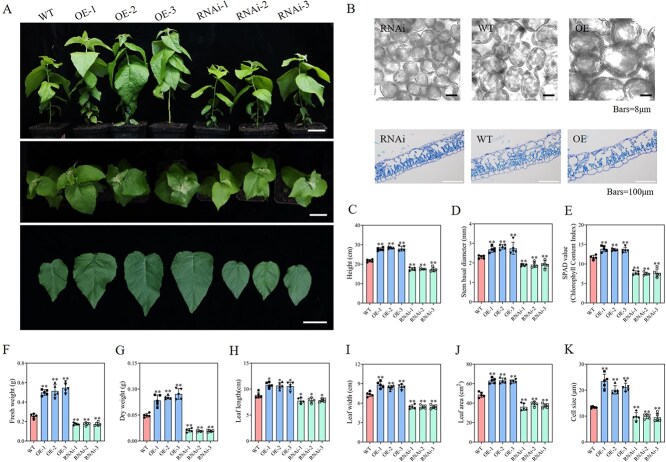
*PpnXTH9* significantly affects poplar cell and leaf size as well as plant growth. (A) Phenotypes of 1-month-old plants and the fifth leaf position of OE and RNAi poplars, bars = 5 cm. (B) Mesophyll and epidermal cells of the fifth leaf of OE and RNAi lines; (C) plant height; (D) stem basal diameter; (E) SPAD value; (F) leaf fresh weight; (G) dry weight; (H) leaf length; (I) leaf width; (J) leaf area; and (K) cell size. Values represent the mean ± SD (*n* ≥ 3). ^*^P < 0.05, ^**^P < 0.01.

By observing the subcellular localization of PpnXTH9, it was found that it was mainly located on the plasma membrane (Fig. S15). To verify the function of *PpnXTH9* in poplar, *PpnXTH9* OE and RNAi lines were constructed. The results showed that 1-month-old OE plants had significant growth advantages compared with WT plants; their plant height, basal diameter, leaf SPAD value, leaf fresh weight and dry weight, leaf length, leaf width, and leaf area all increased significantly. The RNAi plants showed the opposite changes, which were significantly lower than those of WT plants (Fig. 7A, C–J). The mesophyll and leaf epidermal cells of OE plants were significantly larger than those of WT plants, while the mesophyll and leaf epidermal cells of RNAi plants were significantly smaller than those of WT plants (Fig. 7B and K).

## Discussion

### Transcription factors GATA8 and GRF5 directly regulate the expression of *XTH9*, which determines plant cell size

Study shows that polyploid plant cells have enormous size [8]. Obviously, the expression of genes related to cell size determination in polyploid plants must be different from that in diploid plants; these DEGs should be the key genes that determine cell size. Through analysis of transcriptome sequencing data of triploid poplar, we found that *PpnGATA8* was upregulated in apical buds of triploid poplar (Fig. 2C), and its gene sequence was highly similar to the GATA8 sequence in *P. trichocarpa* and *A. thaliana* (Fig. S3). *GATA8* belongs to the *GATA* family, which can bind to the (A/T)-GATA-(A/G) sequence in the promoter of the target gene, regulate the transcription level of downstream genes, and affect the growth and development of plants [46]. Studies have shown that *AtGATA8* is involved in *Arabidopsis* cell differentiation and seed germination [38]. OE of *OsGATA8* can significantly improve photosynthetic efficiency and biomass accumulation of rice [39]. Therefore, it is speculated that poplar *PpnGATA8* has similar functions and may regulate plant cell development and biomass accumulation.

**Figure 8:**
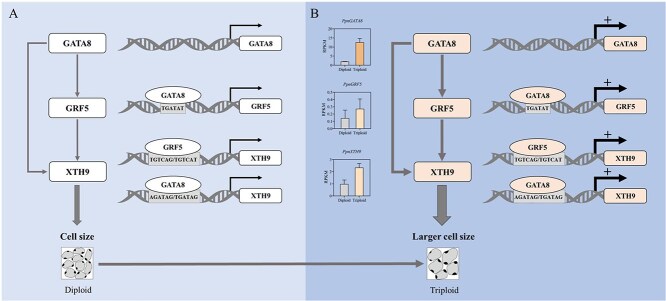
Schematic diagram of the *GATA8*-*GRF5*-*XTH9* module regulating plant cell size. (A) *GATA8* binds to the promoters of *GRF5* and *XTH9*, respectively and promotes the expression of *GRF5* and *XTH9*. Meanwhile, *GRF5* binds to the promoter of *XTH9* and promotes the expression of *XTH9*, thereby affecting plant cell size. (B) Diagram of the transcriptional regulation pattern of leaf cell enlargement in triploid poplars. The histograms next to the gene indicate the RPKM values of the gene in diploid and triploid poplars.

Transgenic lines of *PpnGATA8* were constructed in *Arabidopsis* and poplar, and it was found that OE of *PpnGATA8* could significantly increase plant cell size (Fig. 3; Fig. S4). Data from the *PpnGATA8*-OE transcriptome showed that the expressions of *PagGRF5* and *PagXTH9* were significantly upregulated (Fig. 4H), further proving that *PpnGATA8* can directly regulate the expression of *PagGRF5* and *PagXTH9* (Fig. 5). We further verified that *PpnGRF5* can directly regulate and promote the expression of *PagXTH9*, and when *PpnGATA8* and *PpnGRF5* are present at the same time, its promoting effect on *PagXTH9* was the strongest (Fig. 6I). Therefore, it is proven that *GATA8* is located upstream of *GRF5* and *XTH9*, and *GRF5* is also located upstream of *XTH9*; the three form a feedback regulation pattern (Fig. 8A). *PpnGRF5*-OE and *PpnXTH9*-OE poplars were constructed, and it was found that the cell size of *PpnGRF5*-OE and *PpnXTH9*-OE lines significantly increased (Figs 6A and 7), indicating that the regulatory relationship between *GATA8*, *GRF5,* and *XTH9* is related to plant cell development.

In summary, *GATA8* can directly bind to TGATAT, AGATAG, or TGATAG in the promoter sequences of *GRF5* and *XTH9*, thereby promoting the expression of *GRF5* and *XTH9*; meanwhile, *GRF5* can directly bind to TGTCAG or TGTCAT in the promoter sequence of *XTH9*, thereby promoting the expression of *XTH9*, thus regulating plant cell size (Fig. 8A).

### Coherent FFL regulation pattern formed by GATA8 and GRF5

An FFL is a common gene regulatory network pattern that is widely present in plants and usually has functions such as regulating biological rhythms and development, triggering temporary or pulsed gene expression, stress resistance response, and fine-tuning developmental processes [27, 47]. FFL is divided into coherent FFL (CFFL) and incoherent FFL (IFFL) [48, 49]. In CFFL, the input signal activates the expression of downstream genes through intermediary molecules (usually transcription factors), and the intermediary molecules themselves also promote the expression of downstream genes [48]. In this pathway, the input signal and the output reaction direction are consistent, resulting in an amplification effect of the signal. Our study found that the regulatory relationship between *GATA8*, *GRF5*, and *XTH9* formed a CFFL pattern, in which *GATA8* and *GRF5* had additive effects in regulating *XTH9*. This mode can more flexibly regulate the expression of target genes through multiple upstream factors. In particular, especially when both pathways are activated, the expression of target genes will be enhanced, which helps plants to quickly increase the expression level of target genes when a strong response is needed, thereby enhancing the response effect [50]. Our study found that the expression level of *XTH9* was significantly upregulated in overexpressing *PpnGATA8* and *PpnGRF5* poplars (Fig. 6H). The expression changes of two regulatory factors, *GATA8* and *GRF5*, were consistent with those of functional gene *XTH9*, showing typical CFFL characteristic.

### Transcriptional regulation of cell gigantism in polyploid plant leaves

Polyploid plants tend to have larger cell sizes than diploid plants [51, 52]. Transcriptome sequencing results showed that transcription factors such as *GATA8* and *GRF5*, which influence cell division or differentiation, were significantly highly expressed in triploid poplars (Fig. 8B). Among them, *GRF5* can significantly affect the size of cells in polyploid plants [42].

Our study verified through a series of molecular biological experiments that *GATA8* directly promotes *GRF5* and *XTH9* expression; while *GRF5* directly promotes the expression of *XTH9* (Figs 5 and 6). Compared with the diploid offspring of the same parental combination, triploid poplar leaf cells showed gigantism. The expression levels of transcription factors *GATA8* and *GRF5* and the cell size promoting factor *XTH9* were upregulated in triploid poplars (Fig. 8B). Therefore, we speculate that in the plant cell size regulatory network, the expression of genes such as *GATA8* and *GRF5* shows a dose effect. *GATA8* and *GRF5*, which significantly upregulated expression in triploid poplars, form an FFL pattern with the downstream functional gene *XTH9* that determine cell size. When the two transcription factors have the same regulatory effect on downstream target gene, the promotion of *XTH9* has an additive effect, that is, *XTH9* may have a greater contribution to the cell size of triploid plant leaves under the action of *GATA8* and *GRF5*. The gene expression of triploid plants shows a superposition effect between the two transcription factors under the dosage effect, resulting in a relatively high expression of the cell size promoting factor *XTH9*, which is an important molecular mechanism that determines the formation of cell giantism in triploid plants.

In conclusion, our study preliminarily revealed the reasons why leaf cells in polyploid plants become larger, proposed a new direction for the functional research of genes such as *GATA8*, *GRF5*, *XTH9*, and provided multiple target genes for plant molecular design breeding. It also provided new germplasm materials for breeding plants with extremely large cells and vegetative organs along with high photosynthetic and growth rates.

## Materials and methods

### Materials and growing conditions

Plant materials for phenotypic observation, tissue-specific gene expression assays, and gene cloning were obtained from a cross between the same parents, ‘Zheyin 3 poplar’ and ‘Beijing poplar’, comprising both triploid and diploid populations [53]. The transgenic vector material was derived from 84 K poplar.

*Nicotiana benthamiana* and *A. thaliana* were grown in an artificial climate chamber (25°C, 16 h light per day) and used for subcellular localization, dual-luciferase reporter gene assays, and genetic transformation, respectively.

### Microscopic observation of mesophyll cells

For histological analysis of leaf cells, leaves at different leaf positions were fixed overnight in FAA solution. Wash the leaves with chloral solution. The size of mesophyll cells was observed using a laser confocal microscope (TCS SP8, Leica, Germany).

### Gas exchange analysis

Triploid and diploid poplars grown in a greenhouse for 3 months were measured using a portable photosynthesis meter (Li-Cor 6400; Lincoln, USA). The measured indicators were Pn, Gs, Tr, and WUE (Pn/Tr) in apical buds as well as in the third and fifth leaves of triploid and diploid poplars [54].

### Transcriptome sequencing in diploid and triploid poplars

The apical buds of triploid and diploid poplar obtained from the parents of ‘Zheyin 3 poplar’ and ‘Beijing poplar’ were used as materials. Apical buds of poplar were sequenced to obtain transcriptome datasets. The clean reads were mapped to the *P. trichocarpa* genome. This part of the data was subsequently used to screen DEGs in diploid and triploid poplars.

### Tissue-specific expression of genes

Three tissues were collected from 3-month-old poplars, including apical buds (containing incompletely expanded leaves), third and fifth leaf position leaves. The RNA was extracted using an RNAprep Pure Plant Total RNA Extraction Kit (DP441, Tiangen Biochemical Technology Co., Ltd., Beijing, China). RT-qPCR was performed using a 7500 real-time PCR system. The *Actin* gene (*Potri.001G309500*) was used as the internal control [53].

### Subcellular localization analysis

Four-week-old *N. benthamiana* was used as a test material, 35S::pBI121:GFP and 35S::*PpnGATA8*-GFP were transformed into *Agrobacterium* strains, respectively. Then, a bacterial solution with an OD_600_ value of 0.8–1.0 was injected into the tobacco leaves. After 48 h, the subcellular localization of green fluorescent protein (GFP) protein was observed using laser confocal fluorescence microscopy (TCS SP8, Leica, Germany). The excitation and emission wavelengths used were 488 nm and 500–550 nm, respectively [55].

### Transcriptional activation analysis

The coding sequence (CDS) of *PpnGATA8* was ligated into the yeast expression vector pGBKT7 to construct a recombinant plasmid. Then, the recombinant plasmid pGBKT7-*PpnGATA8* and empty pGBKT7 plasmid were transformed into yeast strain AH109, respectively. The transformed yeast was evenly plated on a selective synthetic dropout (SD) medium without Trp, and positive transformants were screened by colony PCR. The screened positive transformants were inserted into YPDA culture medium for shaking, diluted proportionally, and plated on SD medium without Trp and His or without Trp, His, and Ade to observe its viability [42].

### Construction of plasmid vectors and gene transformation

The CDS of *PpnGATA8* was inserted into the pBI121-GFP and pROKII-RNAi vectors to generate 35S::*PpnGATA8*-GFP-nos and 35S::*PpnGATA8*-Loop-*PpnGATA8*-nos loop constructs [53].

*Agrobacterium*-mediated transformation of 84 K poplar samples was performed following a previously described transformation method [56]. We identified positive transgenic lines by PCR and RT-qPCR and then screened to obtain OE and interference expression lines with different expression levels of *PpnGATA8*, and used for cell size studies, photosynthetic parameter determination, and RNA-seq experiments.

### Paraffin section observation of leaf thickness and cell size

The fifth leaves of 1-month-old WT and transgenic plants were fixed in FAA fixative. Observe leaf thickness and cell size through paraffin sections. The samples were dehydrated through a graded ethanol series (70% ethanol for 0.5 h, 85% ethanol for 1.5 h, 95% ethanol for 1.5 h, and 100% ethanol for 1 h). They were then cleared with xylene and infiltrated with molten paraffin at 60°C. After complete paraffin infiltration, the tissues were embedded in paraffin blocks and sectioned at a thickness of 8 μm using a rotary microtome. The sections were mounted on glass slides, dewaxed in xylene, and rehydrated through a series of ethanol solutions with decreasing concentrations (100%, 95%, 85%, and 70%). Finally, the rehydrated sections were stained with 0.5% toluidine blue. The materials were then placed under a BX51 microscope (Olympus, Tokyo, Japan) for observation and photography. Cell size statistics were performed using Image J 1.52v.

### Determination of hormone content

Leaves of WT and transgenic plants were collected and ground into dry powder in liquid nitrogen, and appropriate amounts of fresh plant leaves were weighed into glass test tubes. All transgenic lines were divided into three biological replicates. Add isopropanol-water-hydrochloric acid mixed extract to a glass test tube. Then, 8 μl of 1 μg/ml internal standard solution was added and shaken at low temperature for 30 min. Then add dichloromethane and take the lower organic phase. Dry the organic phase with nitrogen gas under lightproof conditions. The supernatant was filtered through a 0.22-μm filter and subsequently detected by High-Performance Liquid Chromatography–Tandem Mass Spectrometry (HPLC-MS/MS).

### RNA-seq experiment of transgenic lines and data analysis

The fifth leaf of overexpressed *PpnGATA8* and WT poplars was harvested. For each line, fifth leaf of five plants with similar growth was selected and mixed. Both the OE and WT poplars contained three biological replicates. Transcriptome sequencing was performed by Novogene Co., Ltd. (Beijing, China). Clean reads were mapped to the 84 K polar genome using HISAT2 [57]. The DESeq R package was used for differential gene expression analysis [58].

### Gene ontology and KEGG enrichment analysis

GO and KEGG enrichment analysis of DEGs were performed using the clusterProfiler R package. GO terms with a corrected *P*-value < 0.05 were considered significantly enriched by DEGs. KEGG is a database resource for understanding the high-level functions and utilities of biological systems.

### Yeast one-hybrid assays

The CDSs of *PpnGATA8* and *PpnGRF5* were fused into the activation domain of the pJG4-5 vector to generate the pJG4-5-*PpnGATA8* and pJG4-5-PpnGRF5 constructs. Fragments containing the putative *PpnGATA8* binding site as well as alternative mutants with binding sites in the *PagGRF5* and *PagXTH9* promoters were amplified and fused into the vector pLacZi2μ. pJG4-5-*PpnGATA8* and pLacZi2μ-*PagGRF5*, pJG4-5-*PpnGATA8* and pLacZi2μ-*PagXTH9*, pJG4-5-PpnGRF5 and pLacZi2μ-*PagXTH9* were cotransformed into the EGY48 yeast strain. The yeast was then inoculated on a medium containing X-gal without tryptophan and uracil for incubation and observation [53].

### Electrophoretic mobility shift assays

EMSAs were performed to examine whether PpnGATA8 could bind to the promoters of *GRF5* and *XTH9* and whether *GRF5* could bind to the promoter of *XTH9*. For this purpose, two complementary 60-bp oligonucleotides containing binding *cis*-elements were separately synthesized and labeled with biotin using the EMSA Probe Biotin Labeling Kit (Beyotime, cat GS008). GST, GST-PpnGATA8, and GST-PpnGRF5 recombinant proteins were expressed in the *Escherichia coli* BL21 (DE3) strain and purified using a GST-tag Protein Purification Kit (Beyotime, cat P2262). The DNA gel mobility shift assays were performed using an EMSA Kit (Beyotime, cat GS009) [53].

### ChIP-qPCR assays

ChIP assays were performed using leaves from 1-month-old *PpnGATA8*-OE transgenic and WT poplars. Three independent biological replicates were conducted. For each replicate, leaf tissues were harvested and cross-linked with methanol-free formaldehyde, and the reaction was quenched with glycine. Nuclei were isolated, and chromatin was extracted and sheared by sonication to fragments smaller than 1000 bp. After centrifugation, the supernatant containing fragmented chromatin was collected, and a portion was reserved as the input control. The remaining chromatin was immunoprecipitated with a GFP polyclonal antibody (Thermo Fisher, A-11122), while normal IgG was used as a negative control. Immunoprecipitated chromatin was recovered using Protein A/G magnetic beads, followed by elution and reverse cross-linking. DNA was purified using a gel recovery kit. ChIP-qPCR was performed to determine the enrichment of target genomic regions. Fold Enrichment = 2^(−ΔΔCt), ΔΔCt = ΔCt (ChIP-target) − ΔCt (ChIP-IgG) [53].

### Dual-luciferase assays

The CDSs of *PpnGATA8* and *PpnGRF5* were recombined into the pGreenII62SK vector, and the promoters of *PagGRF5* and *PagXTH9* were inserted into the pGreenII 0800 vector. The plasmid of the recombinant vector and the empty plasmid were transformed into *Agrobacterium tumefaciens* strain GV3101, respectively. Mixed bacterial cultures were coinjected into *N. benthamiana* leaves. After 48 h, leaves were collected and infiltrated with 150 μg/ml fluorescein substrate. Fluorophore activity was detected using the Night SHADE LB 985 system (Berthold, Germany). During the image visualization process, we normalized the luminescence value of the empty carrier control group to zero. The leaves injected with the bacterial solution were triturated and assayed for LUC and REN enzyme activities, and their ratios were calculated [53, 59]. All assays were performed in three biological replicates.

### Protoplast transfection experiments

The leaves of poplar tissue culture seedlings cultured for ~30 days were collected and cut into small pieces (length <0.5 mm), with a total amount of ~5–10 g. Then, add 5–10 ml of enzymatic solution to the leaves to completely soak the tissue. The leaves were enzymatically digested for 3–4 h at 25°C with slow shaking (200 rpm). The protoplasts were filtered through a 300-mesh filter into a 2-ml centrifuge tube, and then centrifuged at 600 rpm for 4 min, and a turbid precipitate was visible. Then, the supernatant was directly removed and the tube was washed twice with 10 ml of precooled W5 solution, centrifuged at 600 rpm for 5 min each time, and a turbid precipitate was visible at the bottom of the tube. Add appropriate amount (0.5–2 ml) of MMG solution to suspend the cells as needed to a concentration of 2 × 10^5^ cells/ml. Take the protoplast suspension (100 μl), DNA (10 μl) and PEG4000 solution (110 μl) and mix them gently. Dilute the protoplasts with 1 ml of W5, mix well to terminate the reaction, and collect the supernatant. Finally, 1 ml of W5 solution was added, and the mixture was cultured at 25°C for 18–24 h. The supernatant was removed, leaving only 100–200 μl of protoplasts. The fluorescence signal was observed under a laser confocal microscope (TCS SP8, Leica, Wetzlar, Germany). Samples with fluorescent signals were collected for RNA extraction and RT-qPCR detection. These assays were performed in three biological replicates. All primers used in the above experiments are listed in Table S9.

### Yeast two-hybrid assay

The CDS of PpnGATA8 was cloned into pGBKT7 to produce a bait vector (pBD- PpnGATA8). The CDS of PpnGRF5 was cloned into pGADT7 to generate a prey vector (pAD-PpnGRF5). Bait and prey vectors were cotransformed into yeast strain Y2HGold to confirm these interactions [60]. The yeast two-hybrid assay was performed using the Matchmaker Gold Yeast Two-Hybrid System (Clontech, USA). The primers are listed in Table S9.

### Split luciferase complementation assay

The CDSs of PpnGATA8 and PpnGRF5 were recombined into vectors (pCambia1300-nLUC and pCambia1300-cLUC) to generate PpnGATA8-nLUC and cLUC-PpnGRF5 fusion plasmids. PpnGATA8-nLUC and cLUC-PpnGRF5 were cotransformed into *N. benthamiana* leaves. Leaves were collected after 3 days and infiltrated with 150 μg/ml fluorescein substrate [60]. The luciferase activity was detected using the Night SHADE LB 985 System (Berthold, Germany). The primers are listed in Table S9.

### Statistical analysis

Statistical analyses were performed using SPSS software (IBM Corp., Armonk, NY, USA) and GraphPad Prism. Significances of differences were analyzed by Student’s *t*-test, one-way analysis of variance (ANOVA), and two-way ANOVA (asterisks denote significant differences).

## Supplementary Material

Web_Material_uhag019

## Data Availability

All study data are included in the manuscript and Supporting Information (Figs S1–S15; Tables S1–S9). RNA-seq data of diploid and triploid poplars reported in this manuscript have been submitted to the CNCB (https://ngdc.cncb.ac.cn/gsa/) with the accession no. CRA027169. RNA-seq data of PpnGATA8 OE lines reported in this manuscript have been submitted to the CNCB with the accession no. CRA027170.
