# Supplementary material for: The GATA8-GRF5-XTH9 feed-forward loop regulates cell size in poplar
Source: Hortic Res. 2026 Jan 20;13(4):uhag019. doi: 10.1093/hr/uhag019 (PMC13103475; doi:10.1093/hr/uhag019)
Supplement: Web_Material_uhag019 [file web_material_uhag019.zip › 3. Supporting Information.docx]

**Supporting Information**

**Figure S1.** Gene expression trend analysis and correlation network construction of diploid and triploid poplars. (A) Gene expression trend analysis of the apical buds and leaves at the 17^th^ and 25^th^ leaf positions in diploid poplars. (B) Gene expression trend analysis of apical buds and leaves at the 17^th^ and 25^th^ leaf positions in triploid poplars. (C) Correlation analysis of gene expression in the apical buds, leaves at the 17^th^ and 25^th^ leaf positions of diploid and triploid poplars. (D) Correlation analysis of gene expression in the apical buds of diploid and triploid poplars.

**Figure S2.** Number of up- and down-regulated genes in DEGs of diploid and triploid poplar.

**Figure S3.** Homologous sequence alignment analysis of PpnGATA8, PtrGATA8, and AtGATA8.

**Figure S4.** Phenotypes of *PpnGATA8*-overexpressing *Arabidopsis thaliana*. (A) cross-sectional view of overexpression (OE) and wild-type (WT) *Arabidopsis* leaves after paraffin sectioning. (B) OE and WT *Arabidopsis thaliana* cell size. Values represent the mean ± SD (*n*=10). *, *P* < 0.05 (determined by Student’s *t*-test).

**Figure S5.** Expression levels of *PpnGATA8* in *PpnGATA8*-OE and *PpnGATA8*-RNAi poplars. (A) The expression of *PpnGATA8* in *PpnGATA8*-OE lines was detected by reverse transcription-quantitative polymerase chain reaction (RT-qPCR). (B) The expression of *PpnGATA8* in *PpnGATA8*-RNAi lines was detected by RT-qPCR. The tissues used were leaves. Values represent the mean ± SD (*n*=3). *, *P* < 0.05; **, *P* < 0.01 (determined by Student’s *t*-test).

**Figure S6.** Leaf biomass statistics of transgenic and WT poplars. Fresh and dry weights of the fifth leaf position of OE and RNAi poplars. Values represent the mean ± SD (*n*=3). *, *P* < 0.05 (determined by Student’s *t*-test).

**Figure S7.** Statistical analysis of leaf cell numbers in transgenic and WT poplars. Number of cells unit area (A) and total number of leaves (B). Values represent the mean ± SD (*n*=5). *, *P* < 0.05 (determined by Student’s *t*-test).

**Figure S8.** Paraffin sections of the fifth internode stem segment from WT, PpnGATA8-OE, and PpnGATA8-RNAi plants.

**Figure S9.** Up- and down-regulated DEGs in OE and WT poplars.

**Figure S10.** 10 genes that were significantly upregulated in OE were randomly selected and detected by RT-qPCR in OE and RNAi poplars. Values represent the mean ± SD (*n*=3). **, *P* < 0.01.

**Figure S11.** Expression of genes related to cell size or plant growth in OE and WT poplars. A heat map shows the FPKM values of some genes in the fifth leaf position.

**Figure S12.** Expression levels of *PpnGATA8* in transgenic plants used to detect *GRF5* and *XTH9* expression levels. Values represent the mean ± SD (*n*=3). **, *P* < 0.01 (determined by Student’s *t*-test).

**Figure S13.** Expression levels of *PpnGRF5* in *PpnGRF5*-OE poplar. Values represent the mean ± SD (*n*=3). **, *P* < 0.01 (determined by Student’s *t*-test).

**Figure S14.** The protein interaction between PpnGATA8 and PpnGRF5 was demonstrated. (A) Yeast cells of co-transformants of PpnGATA8 and PpnGRF5 grown on SD/−Trp-Leu and SD/−Trp-Leu-Ade-His media. PpnGATA8 was fused to the DNA- binding domain (BD), and PpnGRF5 was fused to the transcription activation domain (AD). pAD and pBD were negative controls. (B) Split luciferase complementation assay revealing that there was no interaction between PpnGATA8 and PpnGRF5. PpnGATA8 was fused to the N-terminal portions of LUC (nLUC), and PpnGRF5 was fused to the C-terminal portion of LUC (cLUC). Representative images of *N. benthamiana* leaves 48 h after infiltration are shown.

**Figure S15.** Subcellular localization of the PpnXTH9 protein. Laser-scanning confocal microscopy images of tobacco leaves transiently expressing 35S::GFP and 35S::PpnXTH9-GFP.

**Table S1.** The number of DEGs in diploid and triploid poplars.

**Table S2.** Expression levels of DEGs in diploid and triploid poplar.

**Table S3.** Expression levels of DEGs in OE and WT poplars.

**Table S4.** Gene Ontology (GO) terms of DEGs between OE and WT poplar leaves.

**Table S5.** Kyoto Encyclopedia of Genes and Genomes (KEGG) terms of DEGs between OE and WT poplar leaves.

**Table S6.** Expression levels of genes involved in cell size in OE and WT poplars.

**Table S7.** Cis-elements analysis of the *PagGRF5* promoter. Descriptions and total number of development-related, stress-related, and hormone-responsive cis-elements in the *PagGRF5* promoter region.

**Table S8.** Cis-elements analysis of the *PagXTH9* promoter. Descriptions and total number of development-related, stress-related, and hormone-responsive cis-elements in the *PagXTH9* promoter region.

**Table S9.** Primers used for this research.
