# Supplementary material for: The GATA8-GRF5-XTH9 feed-forward loop regulates cell size in poplar
Source: Hortic Res. 2026 Jan 20;13(4):uhag019. doi: 10.1093/hr/uhag019 (PMC13103475; doi:10.1093/hr/uhag019)
Supplement: Web_Material_uhag019 [file web_material_uhag019.zip › Table S7.docx]

|  | Element | Description | Number |
| --- | --- | --- | --- |
| Development | Box 4 | part of a conserved DNA module involved in light responsiveness | 5 |
|  | G-Box | cis-acting regulatory element involved in light responsiveness | 1 |
|  | G-box | cis-acting regulatory element involved in light responsiveness | 1 |
|  | TCCC-motif | part of a light responsive element | 1 |
|  | GATA-motif | —— | 2 |
| Stress | TC-rich repeats | cis-acting element involved in defense and stress responsiveness | 1 |
|  | LTR | cis-acting element involved in low-temperature responsiveness | 1 |
|  | MBS | cis-acting element involved in drought-inducibility | 1 |
| Hormone | ABRE | cis-acting element involved in the abscisic acid responsiveness | 4 |
|  | CGTCA-motif | cis-acting regulatory element involved in the MeJA-responsiveness | 3 |
|  | P-box | gibberellin-responsive element | 1 |
|  | TGACG-motif | cis-acting regulatory element involved in the MeJA-responsiveness | 3 |

**Table S7.** *Cis*-elements analysis of the *PagGRF5* promoter. Descriptions and total number of development-related *cis*-elements, stress-related *cis*-elements and hormone-responsive *cis*-elements in the *PagGRF5* promoter region.
