# Supplementary material for: The GATA8-GRF5-XTH9 feed-forward loop regulates cell size in poplar
Source: Hortic Res. 2026 Jan 20;13(4):uhag019. doi: 10.1093/hr/uhag019 (PMC13103475; doi:10.1093/hr/uhag019)
Supplement: Web_Material_uhag019 [file web_material_uhag019.zip › Table S8.docx]

|  | Element | Description | Number |
| --- | --- | --- | --- |
| Development | ACE | cis-acting element involved in light responsiveness | 1 |
|  | ATC-motif | part of a conserved DNA module involved in light responsiveness | 1 |
|  | ATCT-motif | part of a conserved DNA module involved in light responsiveness | 2 |
|  | Box 4 | part of a conserved DNA module involved in light responsiveness | 8 |
|  | CAT-box | cis-acting regulatory element related to meristem expression | 2 |
|  | G-Box | cis-acting regulatory element involved in light responsiveness | 2 |
|  | G-box | cis-acting regulatory element involved in light responsiveness | 5 |
|  | GCN4_motif | cis-regulatory element involved in endosperm expression | 1 |
|  | GT1-motif | light responsive element | 1 |
|  | I-box | part of a light responsive element | 1 |
|  | LAMP-element | part of a light responsive element | 1 |
|  | TCT-motif | part of a light responsive element | 1 |
|  | chs-CMA1a | part of a light responsive element | 1 |
|  | circadian | cis-acting regulatory element involved in circadian control | 1 |
|  | TGTCAG-motif | —— | 1 |
|  | GATA-motif | —— | 2 |
| Stress | GC-motif | enhancer-like element involved in anoxic specific inducibility | 1 |
|  | MBS | cis-acting element involved in drought-inducibility | 1 |
| Hormone | ABRE | cis-acting element involved in the abscisic acid responsiveness | 6 |
|  | CGTCA-motif | cis-acting regulatory element involved in the MeJA-responsiveness | 2 |
|  | P-box | gibberellin-responsive element | 1 |
|  | TGACG-motif | cis-acting regulatory element involved in the MeJA-responsiveness | 2 |

**Table S8.** *Cis*-elements analysis of the *PagXTH9* promoter. Descriptions and total number of development-related *cis*-elements, stress-related *cis*-elements and hormone-responsive *cis*-elements in the *PagXTH9* promoter region.
