# Supplementary figures and images for: The GATA8-GRF5-XTH9 feed-forward loop regulates cell size in poplar
Source: Hortic Res. 2026 Jan 20;13(4):uhag019. doi: 10.1093/hr/uhag019 (PMC13103475; doi:10.1093/hr/uhag019)

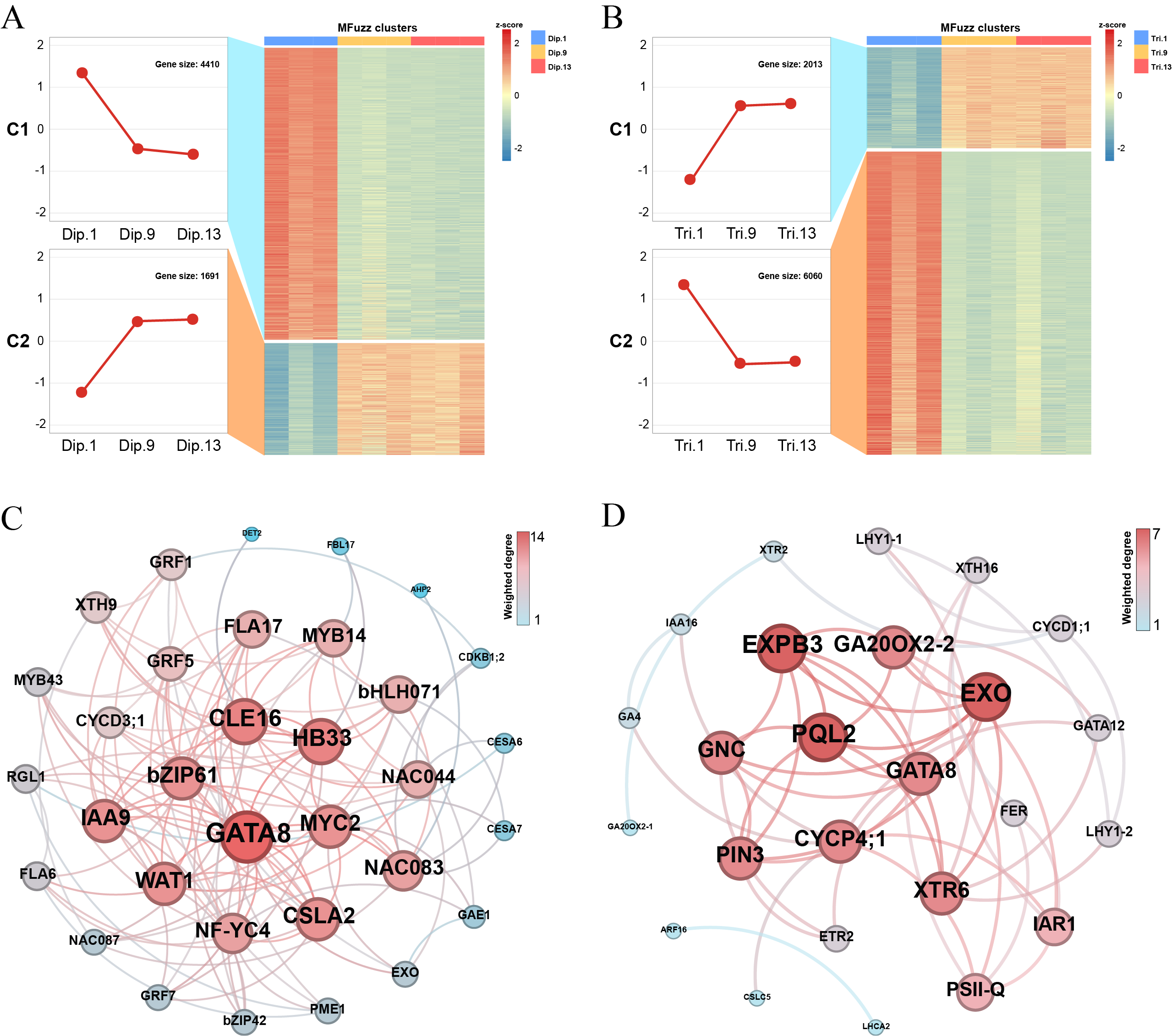

Supplement: Web_Material_uhag019 [file web_material_uhag019.zip › Figure S1.png]

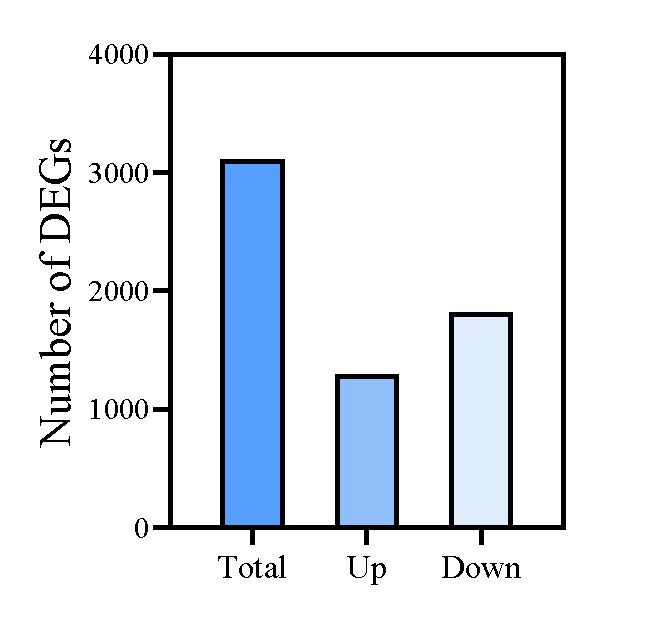

Supplement: Web_Material_uhag019 [file web_material_uhag019.zip › Figure S2.jpg]

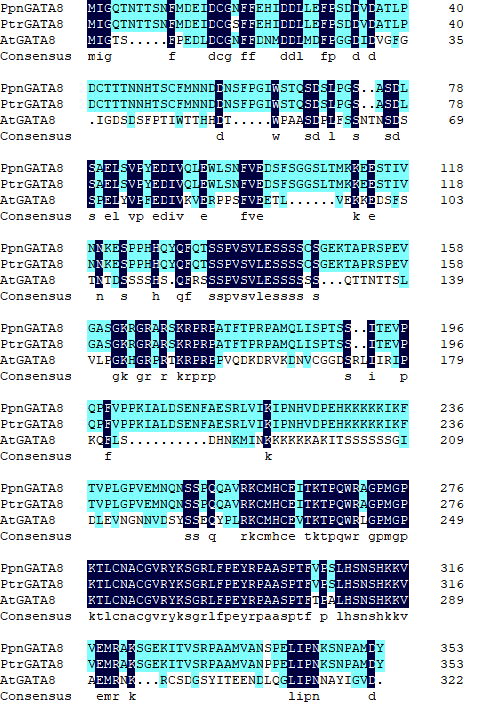

Supplement: Web_Material_uhag019 [file web_material_uhag019.zip › Figure S3.png]

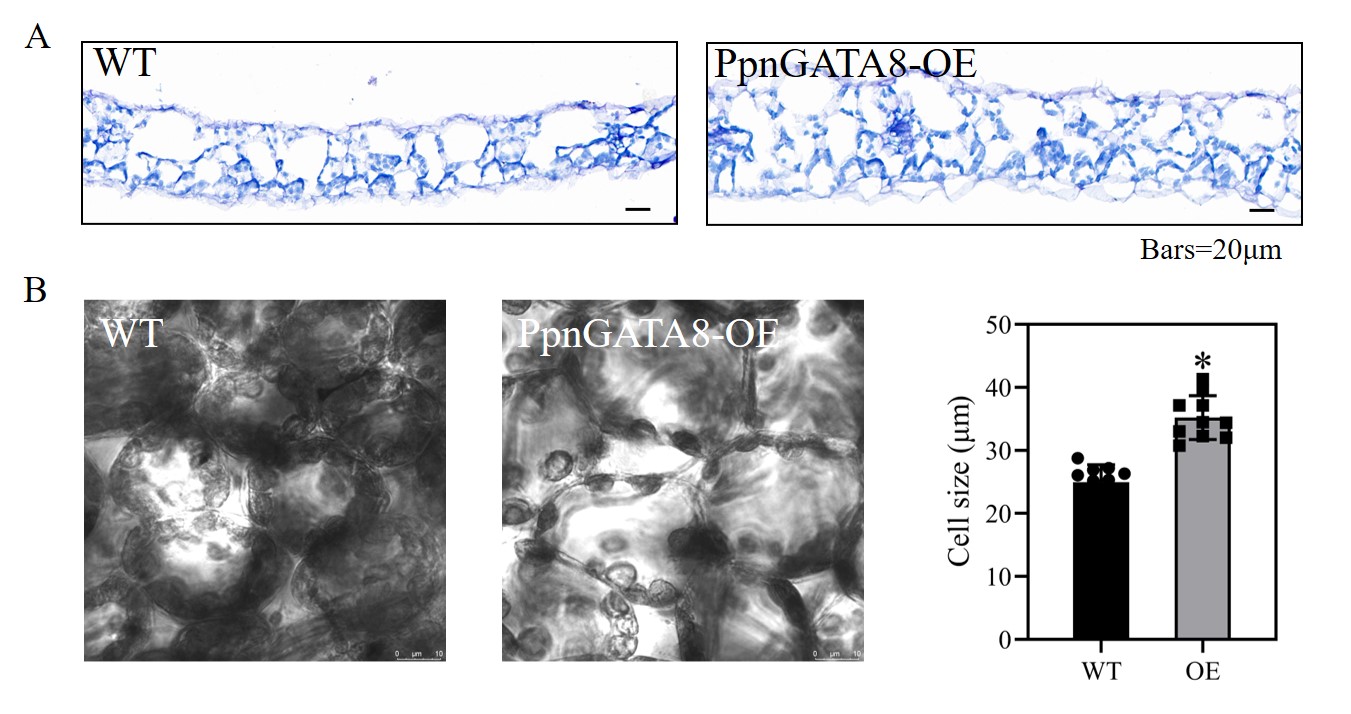

Supplement: Web_Material_uhag019 [file web_material_uhag019.zip › Figure S4.jpg]

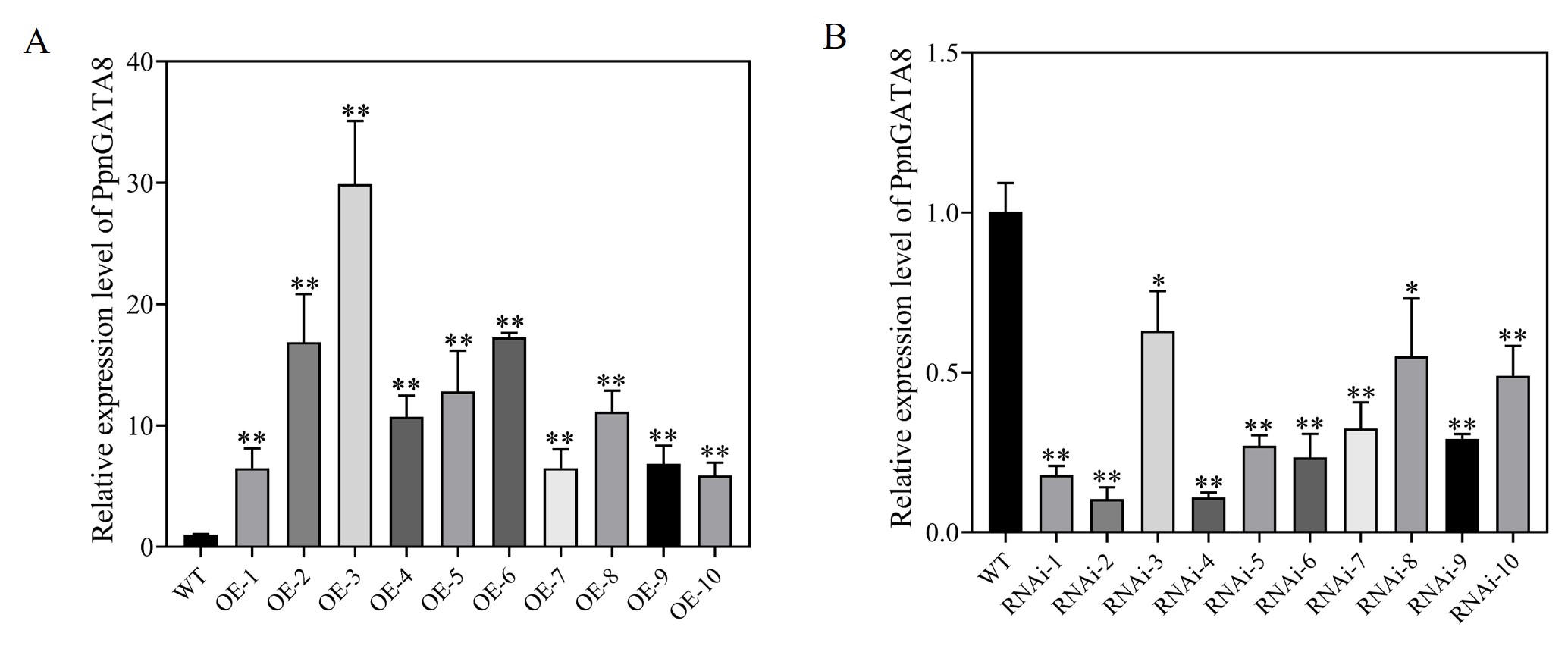

Supplement: Web_Material_uhag019 [file web_material_uhag019.zip › Figure S5.jpg]

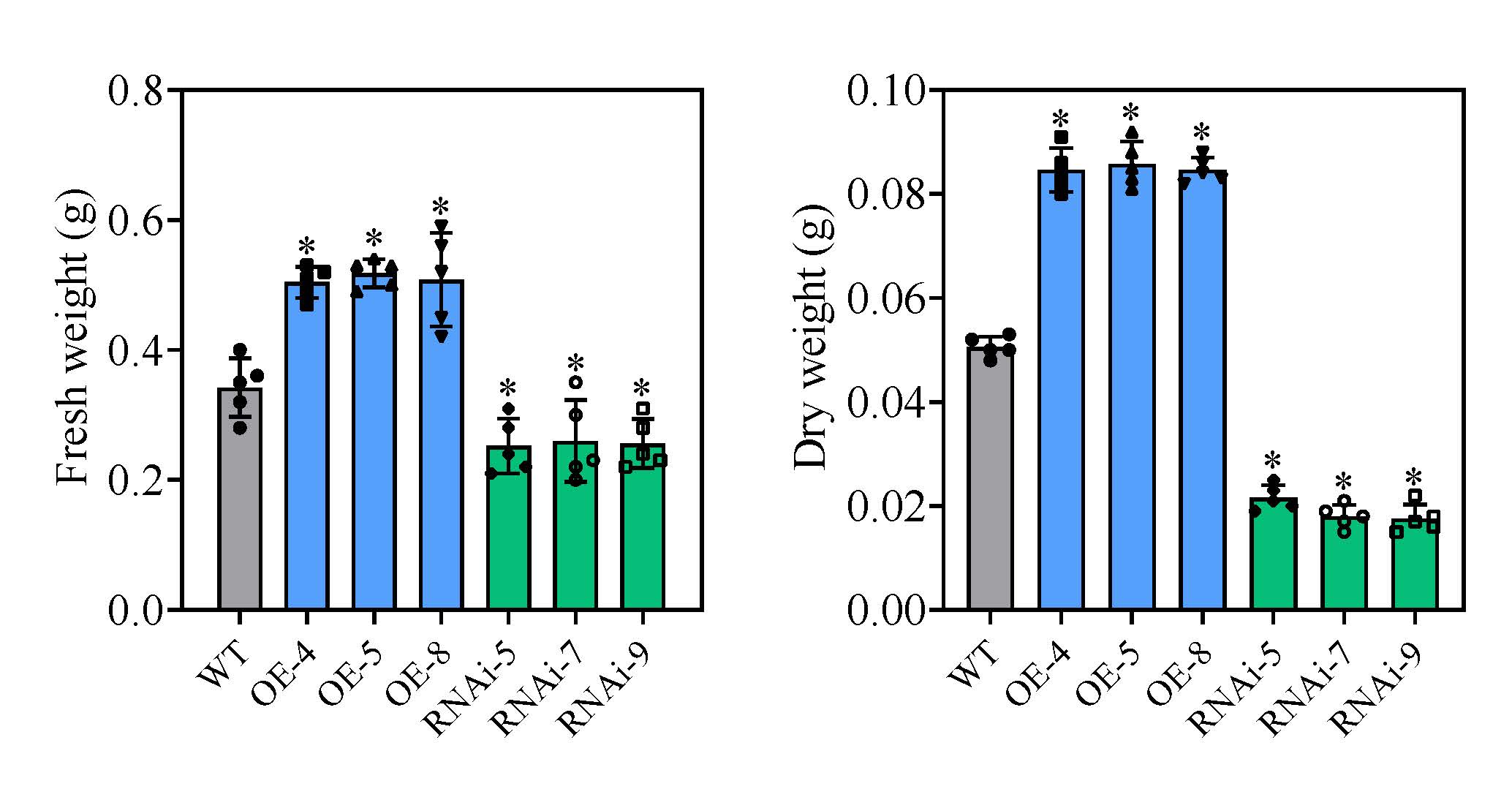

Supplement: Web_Material_uhag019 [file web_material_uhag019.zip › Figure S6.jpg]

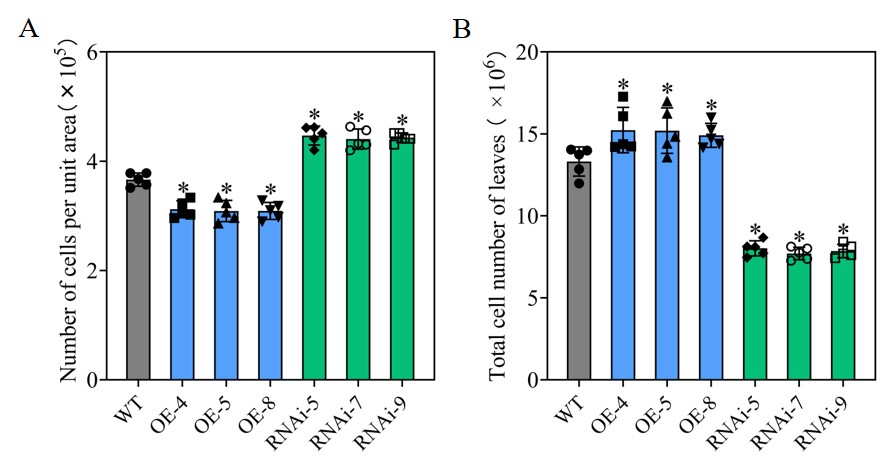

Supplement: Web_Material_uhag019 [file web_material_uhag019.zip › Figure S7.jpg]

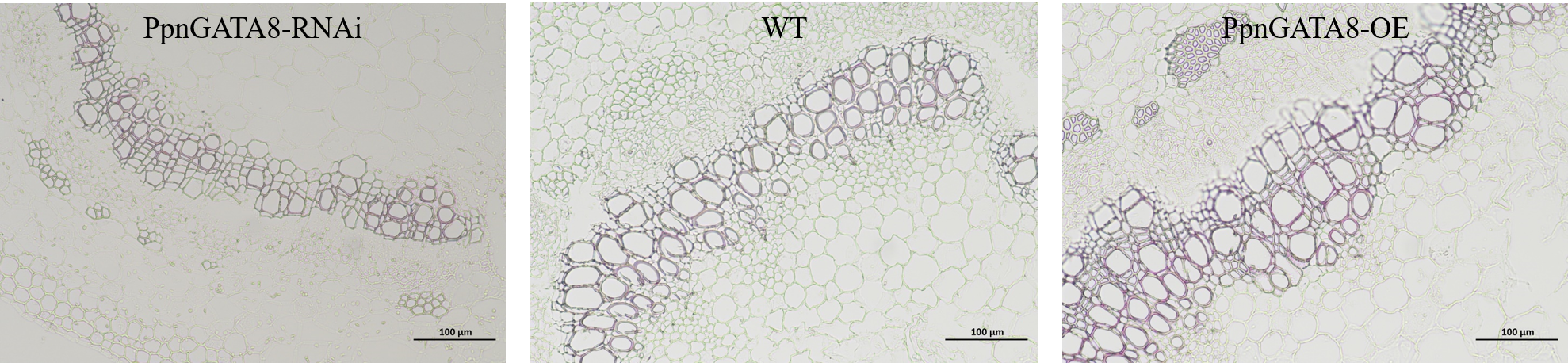

Supplement: Web_Material_uhag019 [file web_material_uhag019.zip › Figure S8.png]

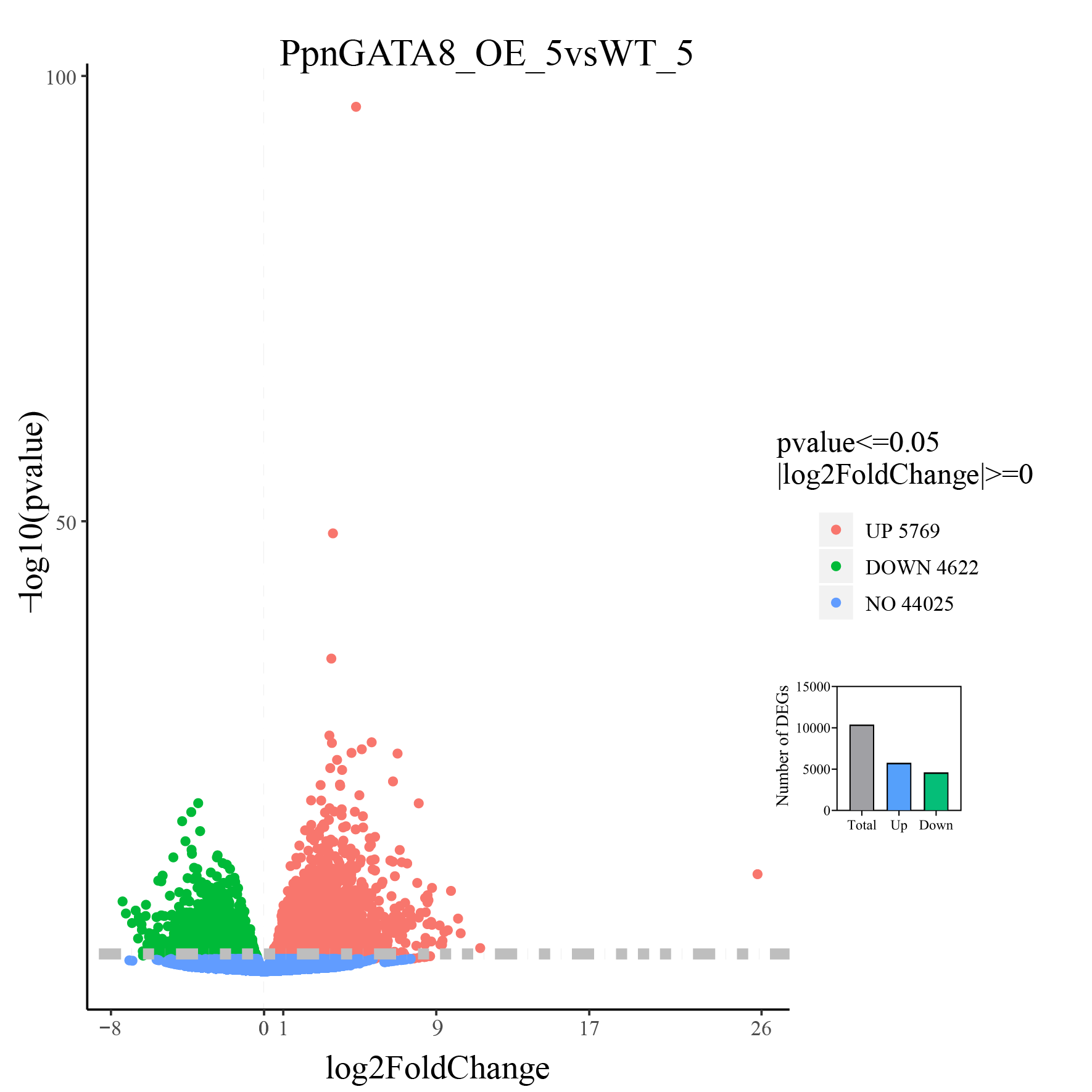

Supplement: Web_Material_uhag019 [file web_material_uhag019.zip › Figure S9.png]

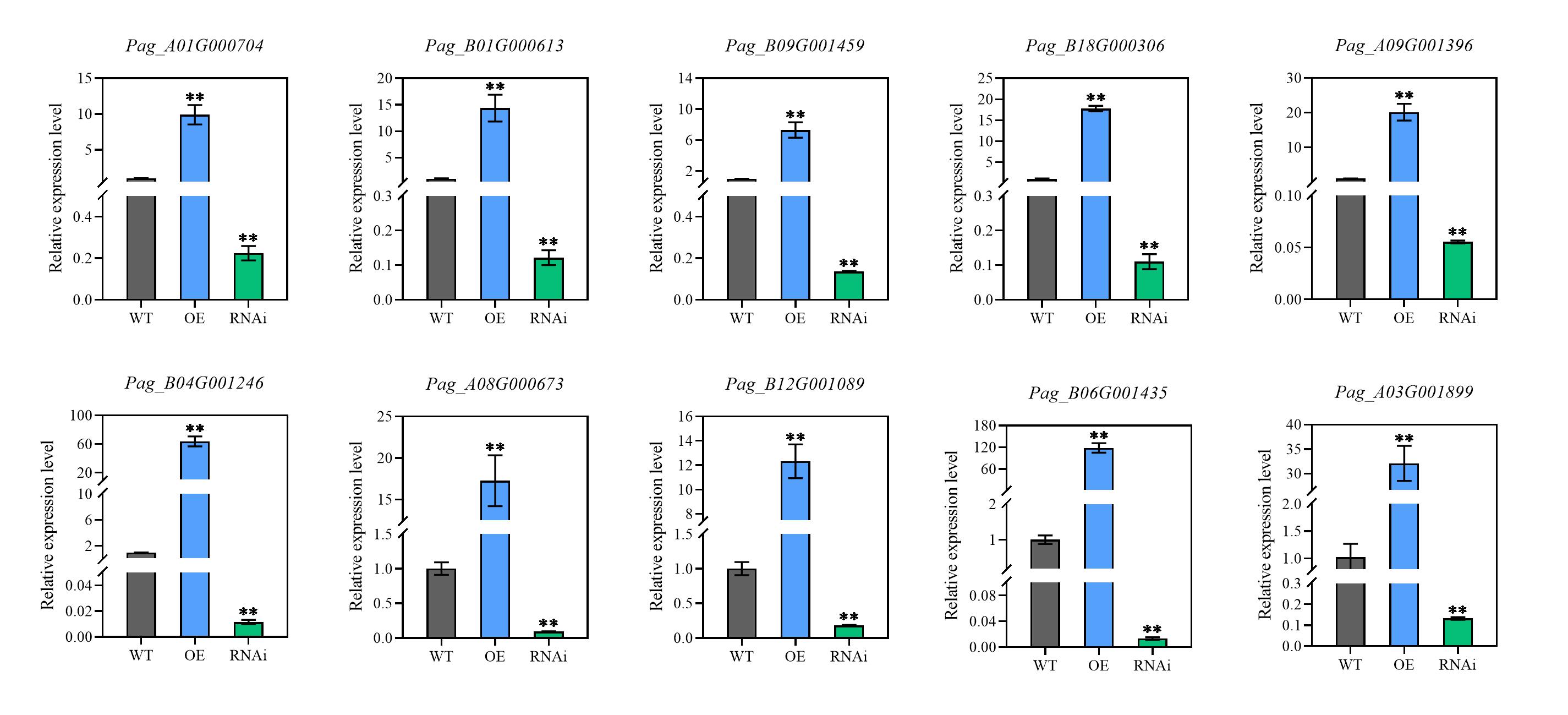

Supplement: Web_Material_uhag019 [file web_material_uhag019.zip › Figure S10.jpg]

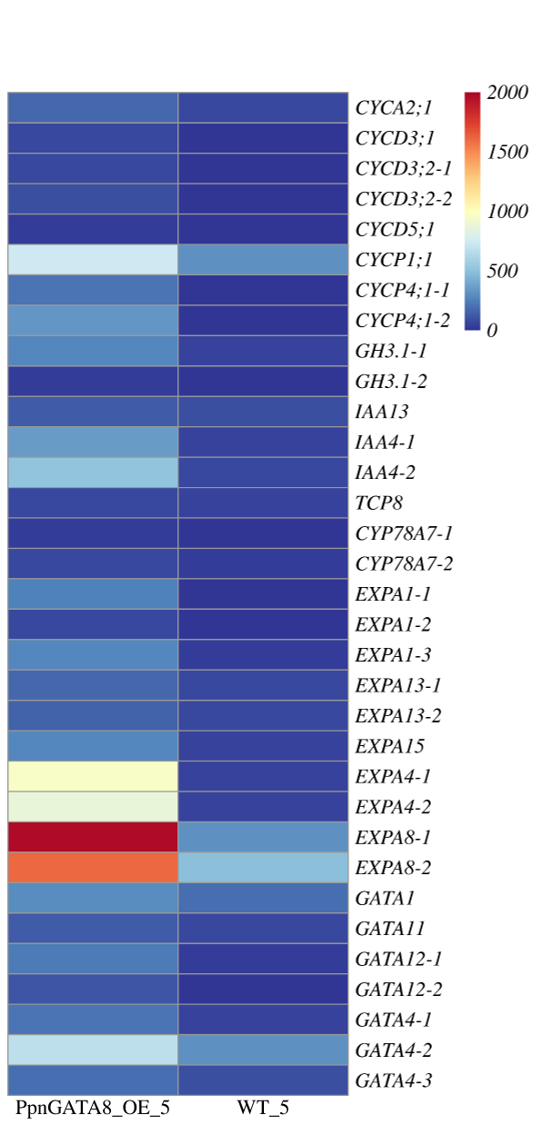

Supplement: Web_Material_uhag019 [file web_material_uhag019.zip › Figure S11.tif]

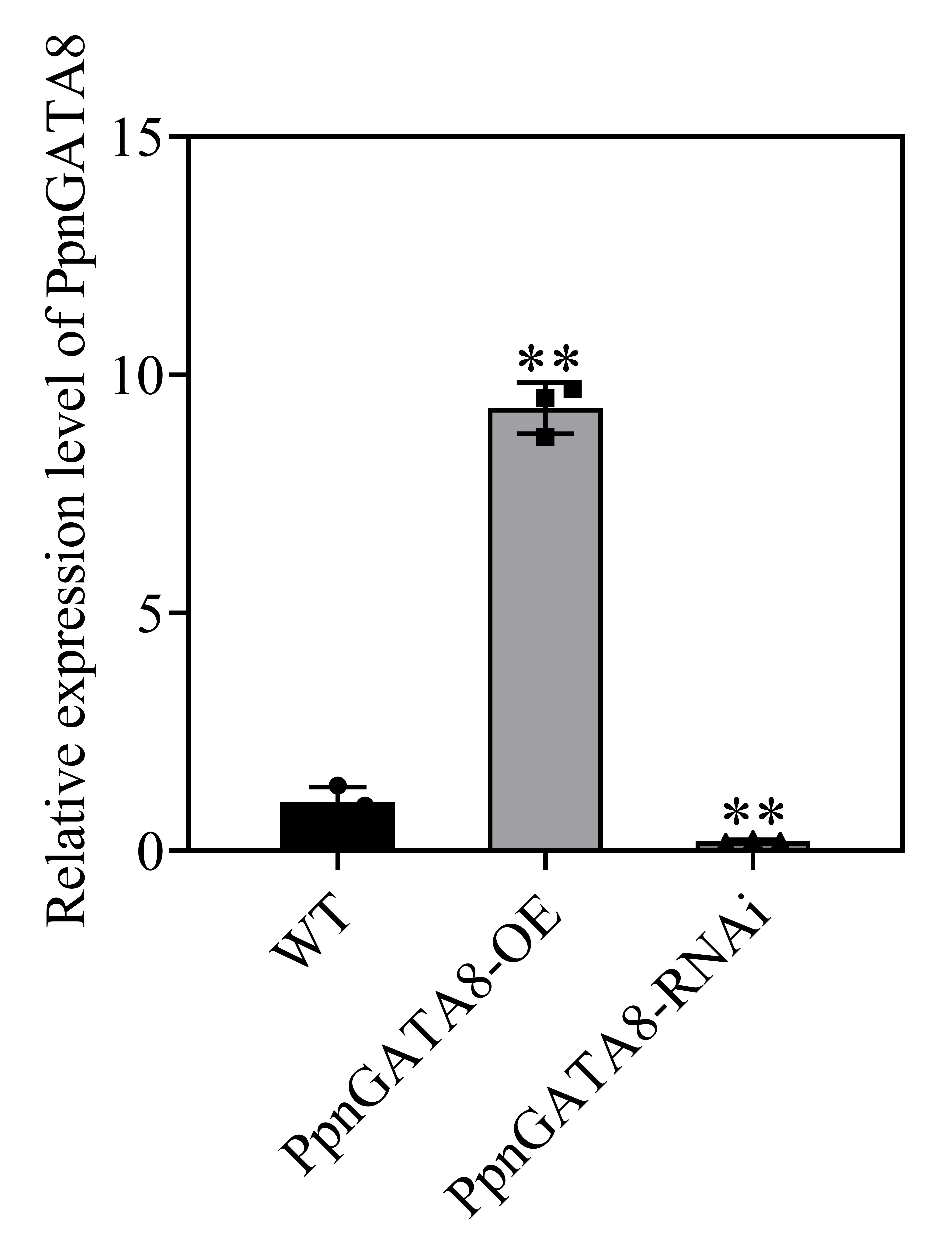

Supplement: Web_Material_uhag019 [file web_material_uhag019.zip › Figure S12.jpg]

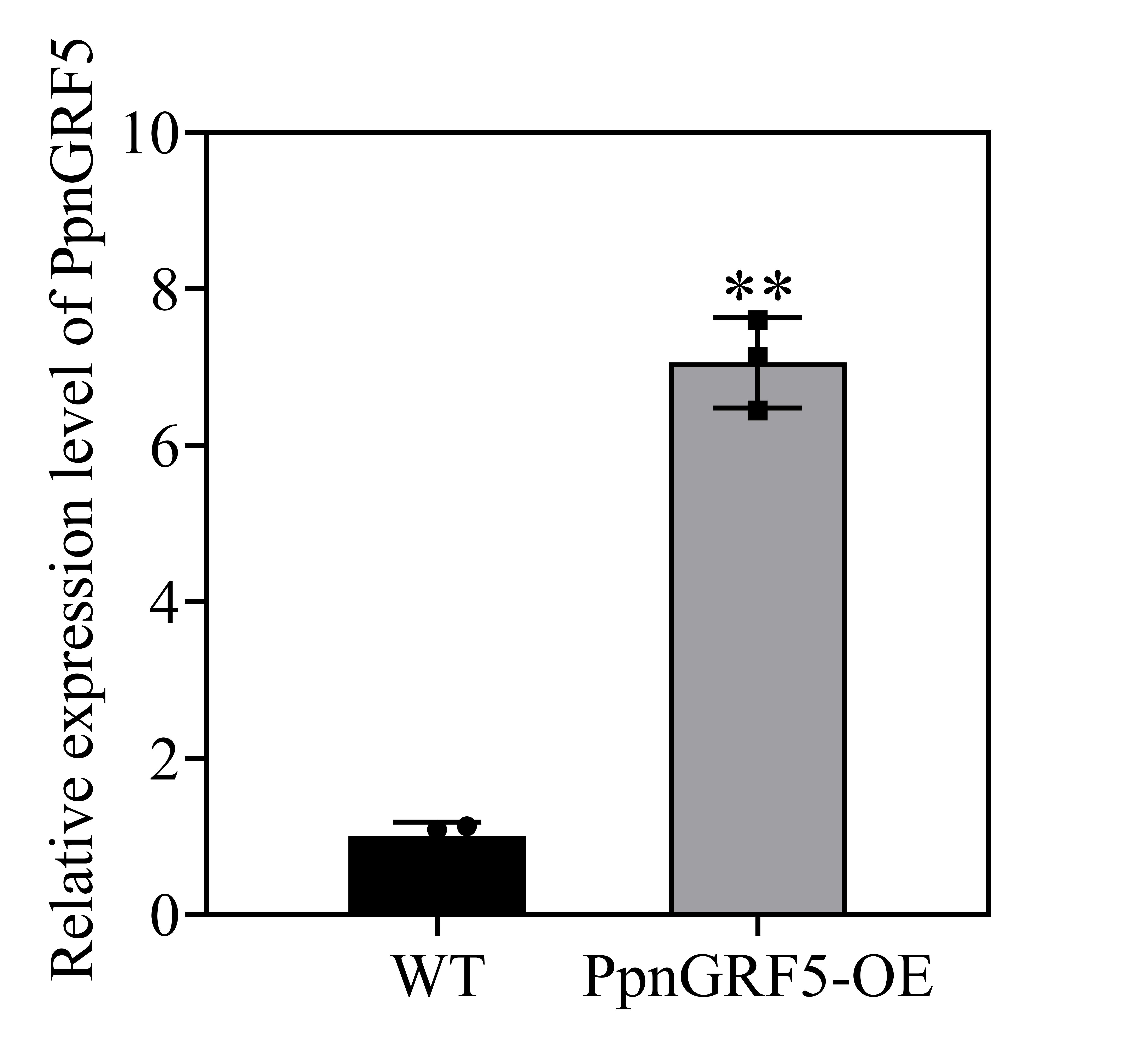

Supplement: Web_Material_uhag019 [file web_material_uhag019.zip › Figure S13.jpg]

A

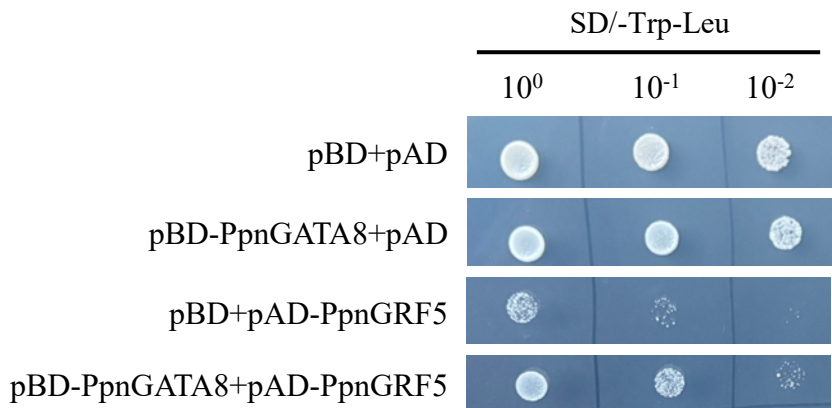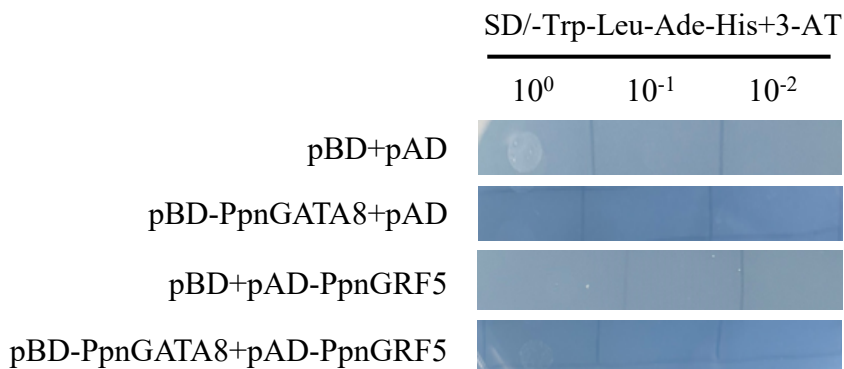

B

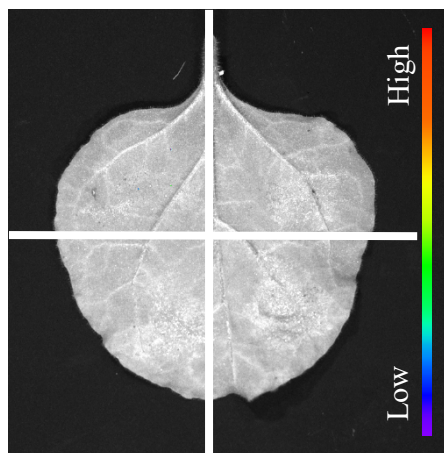

|                                        |                            |
|----------------------------------------|----------------------------|
| nLUC<br>+cLUC                          | PpnGATA8-<br>nLUC<br>+cLUC |
| PpnGATA8-<br>nLUC<br>+cLUC-<br>PpnGRF5 | nLUC<br>+cLUC-<br>PpnGRF5  |

Supplement: Web_Material_uhag019 [file web_material_uhag019.zip › Figure S14.pdf]

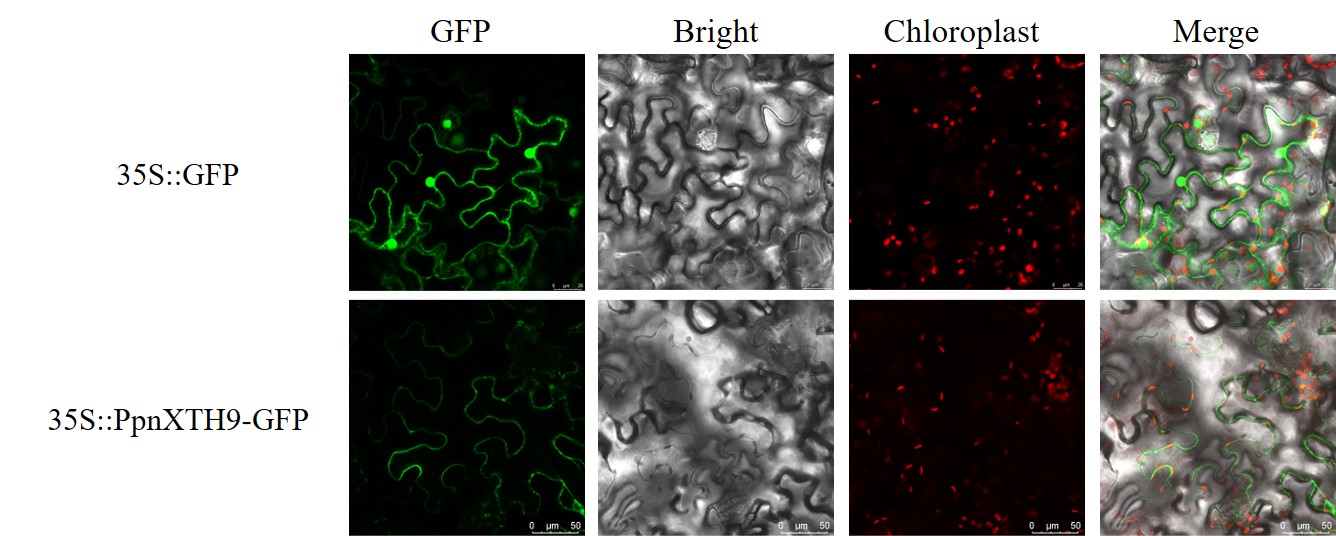

Supplement: Web_Material_uhag019 [file web_material_uhag019.zip › Figure S15.jpg]
